# Mitochondrial supercomplex assembly regulates metabolic features and glutamine dependency in mammalian cells

**DOI:** 10.7150/thno.78292

**Published:** 2023-05-21

**Authors:** Kun Zhang, Linjie Chen, Bo Wang, Deyu Chen, Xianglai Ye, Xinyu Han, Quan Fang, Can Yu, Jia Wu, Sihan Guo, Lifang Chen, Yu Shi, Lan Wang, Huang Cheng, Hao Li, Lu Shen, Qiongya Zhao, Liqin Jin, Jianxin Lyu, Hezhi Fang

**Affiliations:** 1 Key Laboratory of Laboratory Medicine, Ministry of Education, Zhejiang Provincial Key Laboratory of Medical Genetics, Department of Cell Biology and Medical Genetics, College of Laboratory Medicine and Life sciences, Wenzhou Medical University, Wenzhou 325035, China.; 2 Department of Clinical Laboratory, Xi'an Daxing Hospital, Xi'an 710016, China.; 3 Department of Laboratory Medicine, Zhejiang Provincial People's Hospital, Affiliated People's Hospital of Hangzhou Medical College, Hangzhou 310000, China.; 4 School of Laboratory Medicine and Bioengineering, Hangzhou Medical College, Hangzhou 310053, China.; 5 Key Laboratory of Biomarkers and In vitro Diagnosis Translation of Zhejiang province, Zhejiang, Hangzhou 310063, China.

**Keywords:** SCAF1, COX7A2L, mitochondrial supercomplex, metabolism, PDAC

## Abstract

**Rationale:** Mitochondria generate ATP via the oxidative phosphorylation system, which mainly comprises five respiratory complexes found in the inner mitochondrial membrane. A high-order assembly of respiratory complexes is called a supercomplex. COX7A2L is a supercomplex assembly factor that has been well-investigated for studying supercomplex function and assembly. To date, the effects of mitochondrial supercomplexes on cell metabolism have not been elucidated.

**Methods:** We depleted COX7A2L or Cox7a2l in human and mouse cells to generate cell models lacking mitochondrial supercomplexes as well as in DBA/2J mice as animal models. We tested the effect of impaired supercomplex assembly on cell proliferation with different nutrient supply. We profiled the metabolic features in *COX7A2L^-/-^* cells and *Cox7a2l^-/-^* mice via the combined use of targeted and untargeted metabolic profiling and metabolic flux analysis. We further tested the role of mitochondrial supercomplexes in pancreatic ductal adenocarcinoma (PDAC) through PDAC cell lines and a nude mouse model.

**Results:** Impairing mitochondrial supercomplex assembly by depleting COX7A2L in human cells reprogrammed metabolic pathways toward anabolism and increased glutamine metabolism, cell proliferation and antioxidative defense. Similarly, knockout of *Cox7a2l* in DBA/2J mice promoted the use of proteins/amino acids as oxidative carbon sources. Mechanistically, impaired supercomplex assembly increased electron flux from CII to CIII/CIV and promoted CII-dependent respiration in *COX7A2L^-/-^* cells which further upregulated glutaminolysis and glutamine oxidation to accelerate the reactions of the tricarboxylic acid cycle. Moreover, the proliferation of PDAC cells lacking COX7A2L was inhibited by glutamine deprivation.

**Conclusion:** Our results reveal the regulatory role of mitochondrial supercomplexes in glutaminolysis which may fine-tune the fate of cells with different nutrient availability.

## Introduction

Mitochondria generate over 90% of the ATP produced in mammalian cells via two tightly coupled processes carried out by electron transfer chain (ETC) complexes (complex I-IV, CI-IV) and ATP synthase (complex V, CV). Mitochondrial respiration regulates the balance between catabolic and anabolic processes and controls the cell proliferation by fine-tuning the carbon source oxidation 1. Increased mitochondrial retrograde signals such as reactive oxygen species (ROS) 2, and NADH redox state 3 in ETC-deficient cells were found to be sufficient for promoting cell proliferation. Recent studies have suggested that CIII respiration is required for cell proliferation by maintaining the high tricarboxylic anabolic metabolite levels 4, 5. In addition to CIII, other ETC complexes are also essential for cell proliferation as they maintain tricarboxylic acid cycle (TCA)-derived aspartate level 6. While all TCA intermediates seem important for cell survival and proliferation owing to their role in either metabolic or non-metabolic signaling, a persistent TCA cycle rather than a sectional promotion or inhibition of TCA flux is likely preferred in proliferating cells 7. Multiple carbon sources can fuel the TCA, of which glucose generates the highest NADH/FADH_2_ ratio while fatty acids and glutamine generate relatively lower NADH/FADH_2_ ratio 8. Intact ETC is essential for driving oxidative TCA cycling by oxidizing NADH and FADH_2_. The CoQ redox status has been recognized as a key metabolic sensor for fine-tuning ETC signature required to match varied nutrient availability 9. However, how ETC complexes are regulated to co-ordinate the TCA cycle and drive cancer cell proliferation in transformed KRAS mutant cells, which favor glutamine as a major carbon source to fuel TCA, is unclear.

Mitochondrial supercomplexes are molecular structures consisting of an assembly of complexes I, III, and/or IV. Functional supercomplexes constituting CI+CIII+CIV have been experimentally documented 10. Although the substrate channeling model of the supercomplex has been challenged by several kinetics-based studies 11-13, the biological functions of this structure may form a coenzyme Q pool and a cytochrome c pool for efficient electron transfer via coenzyme Q and cytochrome *c*, respectively 10, 14, 15. Irrespective of the substrate channeling model, both the assembly and activity of individual ETC complexes are regulated by the assembly and stability of supercomplexes 16, 17. While changing the function of ETC complexes can reprogram metabolic pathways such as glycolysis 3, de novo pyrimidine synthesis 5, and glutathione metabolism 18, it is plausible to consider that supercomplex assembly is a major regulator of TCA flux and other cellular metabolic processes via its critical role in the activity of ETC complexes. The physiological relevance of mitochondrial supercomplexes in metabolic control is supported by the fact that exercise in human skeletal muscle promotes supercomplex assembly 19, whereas diabetes impairs this assembly 20. Moreover, the regulation of supercomplex assembly may also be associated with metabolism in adipocytes and cancer cells 21. However, whether and how supercomplexes regulate cell metabolism remains largely unknown.

COX7A2L (also referred to as SCAF1) is the only known supercomplex assembly factor. It is required for the assembly of CIII_2_+CIV and CI+CIII_2_+CIV 22, 23, also referred to as Q-respirasome and N-respirasome, respectively 14. Recent studies further suggest that the loss of COX7A2L leads to the impaired assembly of CIII_2_+CIV 24, but an unstable N-respirasome (“respirasome” was used here after, unless otherwise indicated) can still be formed, wherein CIII and CIV are bound independently to CI without interacting with each other 14, 24. The loss of *COX7A2L* leads to diabetes-like phenotypes in mice 25 and to fat accumulation in zebrafish 26. Further, the overexpression of *COX7A2L* leads to metabolic alterations and increased hypoxia tolerance in breast cancer cells during proliferation 27. Despite the role of mitochondrial supercomplex in the regulation of metabolic processes, the depletion of COX7A2L has a minimal effect on the stability of individual ETC complexes CI, CIII, and CIV. This suggests a direct role of mitochondrial supercomplexes in cellular metabolic regulation. COX7A2L-based models are currently the only available models for studying the underlying mechanisms through which mitochondrial supercomplexes regulate the TCA cycle, metabolic remodeling, and nutrient preference.

In this study, we aimed at studying the effect of impaired mitochondrial supercomplex CI+CIIIn+CIVn assembly, by depleting COX7A2L, on cell proliferation. We used targeted and untargeted metabolic profiling, metabolic flux analysis, and transcriptional profiling to study this effect. We revealed that the impairment of mitochondrial supercomplexes by *COX7A2L* depletion reprogrammed metabolic pathways and enhanced glutaminolysis. We further demonstrated that the loss of COX7A2L-mediated supercomplex assembly failure may alternatively force the cells to rely on mitochondrial complex II for respiration, which further boosted glutamine metabolism. Moreover, the physiological relevance of COX7A2L and mitochondrial supercomplex assembly in the regulation of glutaminolysis was highlighted, where the knockdown of *COX7A2L* promoted the proliferation of pancreatic ductal adenocarcinoma cells (PDAC) in a glutamine-dependent manner.

## Materials and Methods

### Human tumor samples

Human PDAC specimens, including 26 paired formalin-fixed, paraffin-embedded sections of primary PDAC specimens and adjacent normal pancreatic tissues (5 μm thick), were obtained from the First Affiliated Hospital of Wenzhou Medical University. This study was approved by the Ethics Committee of Wenzhou Medical University (2017) and performed in accordance with the ethical standards laid down in the 1964 Declaration of Helsinki and its later amendments or comparable ethical standards.

### Animal studies

*Cox7a2l* knockout mice (*Cox7a2l^-/-^*) were purchased from Biocytogen Co. Ltd. (Shanghai, China). Briefly, the gRNA was designed to generate ~3 kb chromosomal deletion (exon 2-4) of the *Cox7a2l* locus on chromosome 17. The following gRNA sequence was used: 5-GCACCGAAAGAGAGTAAGTT-3. Male mice were used in the animal studies. All mice were housed under specific pathogen-free conditions with a 12 h light or dark cycle in the Animal Experimental Center of Wenzhou Medical University. Five-week-old mice (n = 12 per group) were fed with a standard diet obtained from the Animal Experimental Center of Wenzhou Medical University. Body weights were measured weekly during the feeding period. Glucose tolerance test (GTT) and insulin tolerance test (ITT) were performed using a glucometer at 25 and 26 weeks of age, respectively. For GTT, an intraperitoneal injection of glucose (1.5 g/kg) was performed after 13 h of fasting. Blood was then collected from the tail vein at 0, 15, 30, 60, 90, and 120 min post-injection for glucose measurement. For ITT, an intraperitoneal injection of regular human insulin (0.75 IU/kg) was performed after 5 h of fasting. Blood was then collected from the tail vein at 0, 15, 30, 45, and 60 min post-injection. Body composition was analyzed by Echo MRI (Echo Medical Systems, Houston, Texas). Mice were housed in a 16-chamber TSE system (PhenoMaster, TSE Systems, Frankfurt, Germany) for 2 days to calculate whole body metabolism and 7 days to determine the food intake per day through the indirect calorimetry method, following the manufacturer's instructions. For the glutamine diet, 4% glutamine (Sigma-Aldrich, St. Louis, MO, USA) was supplemented in drinking water.

Nude mice (male, 4 weeks) were purchased from Beijing Vital River Lab Animal Technology Co., Ltd. (Beijing, China) and housed in groups in a specific-pathogen-free facility at the Wenzhou Medical University. Nude mice were fed with a standard diet. At five weeks of age, the mice were injected subcutaneously with 5✕10^6^ human pancreatic cancer cells (PANC-1 cell line) under the armpit or in the shoulder. The injected cells were resuspended in a mixture of PBS and Matrigel (Corning, Corning, NY, USA) at a ratio of 1:1. Once the tumors formed, tumor size was measured using a Vernier caliper once every 3-8 days. Tumor volume was calculated according to the following formula: volume = length × width^2^ × 0.5 mm^3^.

All animal care and experimental protocols were in accordance with the recommendations of the Animal Care and Use Committee of Wenzhou Medical University and in compliance with the *Animals in Research: Reporting In vivo Experiments* (*ARRIVE*) guidelines.

### Cell lines and culture conditions

HEK293T, PaTu-8988T, PANC-1, C2C12, and 3T3-L1 cells were purchased from the Cell Resource Center and authenticated by authentication testing (Chinese Academy of Sciences, Shanghai, China). All cells were cultured in high-glucose Dulbecco's modified Eagle's medium (DMEM, Sigma-Aldrich) containing 12% calf serum (Sigma-Aldrich), 100 U/mL penicillin (Beyotime Biotechnology, Shanghai, China), 100 μg/mL streptomycin (Beyotime Biotechnology), and 0.25 μg/mL amphotericin B (Sangon Biotech, Shanghai, China) at 37 °C in a 5% CO_2_ incubator (Thermo Fisher Scientific, Waltham, MA, USA). During metabolic studies, all cells were tested for mycoplasma and declared mycoplasma-free using a MycoAlert™ PLUS detection kit (Lonza Corporation, Basel, Switzerland).

### Construction, transfection, and generation of stable cell lines

pX330-U6-Chimeric_BB-CBh-hSpCas9 (pX330) was obtained from Feng Zhang (Addgene plasmid #42230). To sort the transfected cell clones, the pX330-GFP was constructed by inserting a green fluorescent protein (GFP) coding sequence into pX330. The gRNA was then cloned into pX330-GFP to knockout (KO) *COX7A2L* in 293T, C2C12, and 3T3-L1 cells. The following gRNA sequences were used: 5- TCCACAGTGTATGATTATGC-3 (human) and 5-TGGTGGTCCGGTAAAGCATT-3 (mouse). Cells were transfected with gRNA-containing PX330-GFP with Lipofectamine™ 3000 transfection reagent (Thermo Fisher Scientific), and the stable *COX7A2L*-KO cell line was selected by the limiting dilution method 28.

For COX7A2L expression in *COX7A2L*^-/-^ 293T cells, human WT (NM_001319036.1) and mutant *COX7A2L* cDNAs (Y73A and 70DEL_VP) were synthesized and correspondingly cloned into pCDH-puro-Myc containing a Myc epitope tag on the C-terminus and pCDH-puro by the Beijing Genomics Institute (BGI Corporation, Shenzhen, China). The lentivirus was packaged and co-transfected into *COX7A2L*^-/-^ 293T with *COX7A2L*-containing pCDH-puro or pCDH-puro-Myc, pMD2.G, and psPAX2 at a ratio of 1:1:2 and then transduced to target cells. Stable cell lines were selected using the limiting dilution method, treated with 2 μg/mL puromycin (Sangon Biotech) and maintained in a regular medium with 1 μg/mL puromycin.

Knockdown of COX7A2L and FLAD1 was conducted by the lentiviral transduction of shRNA containing lentivirus. shRNA was constructed into pLKO.1 and pGIPZ using the following sequence: 5- CCGATTCCACAGTGTATGATT -3 (COX7A2L); 5- AGCTGTTCAGTTCCACTCA -3 (FLAD1). Lentiviral production and transduction were performed as described above.

Transient knockdown of COX7A2L in 8988T and PANC-1 cells was conducted by transfecting cells with small interfering RNA (siRNA) (Thermo Fisher Scientific) using the Lipofectamine™ RNAiMAX transfection reagent (Thermo Fisher Scientific) according to the manufacturer's instructions. The following siRNA sequence for COX7A2L was used: 5-CACUAACUGGAGUUGUUAUUGACUU-3.

### Whole genome sequencing and off-target analysis

*COX7A2L*^-/-^ 293T cells (5✕10^7^) were pelleted and sent to Novogene Corporation (Beijing, China) for whole genome sequencing. Sequencing was performed using Novoseq 6000 (Illumina, San Diego, CA, USA). Off-target analysis was performed using Cas-OFFinder 29.

### Blue native PAGE, SDS-PAGE, immunoblotting, and antibodies

Blue native polyacrylamide gel electrophoresis (BNG) and sodium dodecyl-sulfate polyacrylamide gel electrophoresis (SDS-PAGE) were performed as previously reported 30. Briefly, mitochondria from cultured cells were solubilized with digitonin, a 6:1weight ratio of digitonin mitochondria. Membrane proteins from the tissues of mice were extracted according to the published protocol with the indicated amount of digitonin 30. The protein extracts for SDS-PAGE were prepared using RIPA lysis buffer (Cell Signaling Technology, Danvers, MA, USA). Protein extracts were separated by BNG (3-11% gels) or SDS-PAGE (15% gels) and transferred to 0.22 μm polyvinylidene difluoride membranes (PVDF) (Bio-Rad, Hercules, CA, USA) using a transfer system (Bio-Rad). After the transfer, PVDF membranes were incubated with the following primary antibodies: anti-β-actin (sc-47778; 1:5000, Santa Cruz Biotechnology, Dallas, TX, USA), anti-COX7A2L (ab170696; 1:500, Abcam, Cambridge, MA, USA), anti-TOM70 (14528-1-AP; 1:1000, c, Wuhan, Hubei, China), anti-Grim19 (ab110240; 1:1000, Abcam), anti-NDUFB6 (ab110244; 1:1000, Abcam), anti-SDHA (ab14715; 1:1000, Abcam), anti-UQCRC2 (ab14745; 1:1000, Abcam), or anti-ATP synthase subunit-α (ab14748; 1:1000, Abcam). The secondary antibodies used were alkaline phosphatase-conjugated anti-mouse IgG (#7056; 1:2000, Cell Signaling Technology), horseradish peroxidase-conjugated anti-rabbit/mouse IgG (#7074/#7076; 1:2000, Cell Signaling Technology), and fluorescent dye conjugated anti-rabbit/mouse IgG (#5366/5267; 1:2000, Signaling Technology). After incubation with primary and secondary antibodies, signals of BNG were detected with 10 mg/mL 5-bromo-4-chloro-3ʹ-indolyl phosphate p-toluidine (Thermo Fisher Scientific) mixed with 20 mg/mL nitrotetrazolium blue chloride (Sigma-Aldrich). Signals of SDS-PAGE were detected with the Super Signal West Pico chemiluminescent substrate (Thermo Fisher Scientific). For fluorescence signal, membranes were scanned with Odyssey CLX (LI-COR, Lincoln, NE, USA) as previously described 31.

### Measurement of mitochondrial respiration

Complex-dependent mitochondrial respiration was measured using a Clark-type oxygen electrode (OROBOROS, Innsbruck, Austria) as previously described 32. Briefly, approximately 0.5 million cells were permeabilized with 0.02 mg/ml digitonin (Sigma-Aldrich) and added into the chamber with substrate-free respiration buffer. After recording the baseline, CI-, CII-, and CI+CII-dependent respiration were measured by adding malate (1.6 mM) + glutamate (8 mM), succinate (4 mM), or malate (1.6 mM) + glutamate (8 mM) + succinate (4 mM), respectively. Rotenone (0.8 μM, Sigma-Aldrich), 3-nitropropionic acid (3-NP, 12.6 mM, Sigma-Aldrich), and rotenone (0.8 μM) + 3-NP (12.6 mM, Sigma-Aldrich) were used as inhibitors for CI-, CII-, and CI+CII- dependent respiration, respectively. All respiration data were normalized with cell numbers.

### ATP, lactate, and hydrogen peroxide determination

Total ATP and mitochondrial ATP level were measured using an ATP measurement kit (Thermo Fisher Scientific). Briefly, cells were incubated with 1 ml of the record buffer (156 mM NaCl; 3 mM KCl; 2 mM MgSO_4_; 1.25 mM KH_2_PO_4_; 2 mM CaCl_2_; 20 mM HEPES) with or without 5 mM 2-DG and 5 mM sodium pyruvate at 37 °C in a 5% CO_2_ incubator for 2 h. These cells were then washed twice with PBS and measured according to the manufacturer's instructions. Lactate from 48 h culture medium was filtered with a 10 kDa ultrafiltration centrifugation tube (Sigma-Aldrich) and measured using an Amplite™ Fluorimetric L-Lactate assay kit (AAT Bioquest company, CA, USA) according to the manufacturer's instructions. Cellular hydrogen peroxide (H_2_O_2_) was measured using a hydrogen peroxide assay kit (Beyotime Biotechnology) according to the manufacturer's instructions. The luminescence generated by ATP, fluorescence-labeled lactate and absorbance changed by H_2_O_2_ were monitored at 560 nm, excitation/emission of 540/590 nm, and at 560 nm using a SpectraMax iD3 Reader (Molecular Devices), respectively. Relative values were normalized with cell numbers.

### Immunofluorescence and transmission electron microscopy

For immunofluorescence microscopy, cells were cultured on coverslips and fixed with PBS containing 4% paraformaldehyde (pH = 7.40) for 20 min. These cells were permeabilized with PBS containing 100 μM digitonin (Sigma-Aldrich) for 3 min and incubated overnight with anti-TOMM20 (ab78547; 1:250, Abcam). The cells were then incubated with anti-rabbit IgG-Alexa Fluor 594 (#8889; 1:300; Cell Signaling Technology) for 2 h in the dark. After mounting coverslips with an antifade mounting medium (Beyotime), images were captured by a confocal laser-scanning microscope (Nikon Corporation, Tokyo, Minato-ku, Japan) and analyzed with Image-Pro Plus 6.0 (Media Cybernetics, Rockville, MD, USA) 33. Fragmented and tubular mitochondria were counted according to a published method 34.

For transmission electron microscopy, cells were cultured in 100 mm dishes and fixed with 2.5% glutaraldehyde (Shanghai Lanji Technology Development Co., Shanghai, China) for 2 h at 4 °C. Cells were then washed twice with PBS and fixed in 1% tannic acid (SPI Supplies, West Chester, PA, USA) for 1 h in the dark. After washing twice with PBS, cells were stained with 1% uranyl acetate (The 404 Company Limited, China National Nuclear Corporation, Lanzhou, China) for 1 h and dehydrated in ethanol. Finally, specimens were sent to the electron microscope laboratory of the Wenzhou Medical University for analysis. The cristae lumen widths of mitochondria were quantified as previously reported 35.

### Cell proliferation

Cells (1 × 10^5^) were seeded in 6-well plates and cultured in a nutrient-conditioned medium (**Table S1**) with or without 5 mM N-acetyl-L-cysteine (NAC), 2 mM 3-NP, 5 μM phenazine methosulfate (PMS) (all from Sigma-Aldrich), or 50 μM carboxin (MedChem Express, New Jersey, USA) as previously described 36-38. Cell numbers were counted manually using NovoCyte flow cytometry (Agilent Technologies Inc., Santa Clara, CA, USA).

### Apoptosis assay

Cell apoptosis was determined using the FITC Annexin V apoptosis detection kit (BD Bioscience, Franklin, New Jersey, USA). Briefly, 1 × 10^6^ cells were incubated with 5 μM propidium iodide (PI) and 5 μM Annexin V for 15 min in the dark, and then washed twice with the binding buffer. Cells were analyzed by NovoCyte flow cytometry (Agilent Technologies Inc.) to distinguish between viable and apoptotic cells.

### Measurement of mitochondrial ROS

Mitochondrial ROS levels were measured using a MitoSOX™ red mitochondrial superoxide indicator (Thermo Fisher Scientific) according to the manufacturer's instructions. Briefly, approximately 1 × 10^6^ cells were treated with 5 μM indicator for 10 min at 37 °C and washed three times with warm Hank's balanced salt solution (HBSS buffer). The fluorescence at an excitation/emission of 510/580 nm was measured using NovoCyte flow cytometry (Agilent Technologies Inc.) Relative values were normalized with cell numbers.

### Untargeted metabolic profiling and bioinformatics analysis

Cells were cultured for at least 48 h. Approximately 1 × 10^7^ cells were collected and quickly frozen in liquid nitrogen. For mouse tissue, skeletal muscle > 100 mg) was cut at the same anatomic site and frozen in liquid nitrogen. Samples were sent to Metabolon/Calibra Corporation (Hangzhou, Zhejiang, China) for untargeted metabolic profiling. Data were collected in full scan mode and normalized with cell numbers. For metabolic analysis, raw area counts were normalized, and missing values were imputed as the minimum value. Outliers that were defined by values higher than 1.5 times the interquartile range from the quartile computed from *Tukey*'s Hinge were excluded in the statistical analyses. Principal component analysis (PCA) was conducted on the normalized data using MetaboAnalyst 4.0 to determine the reproducibility of the experiments 39. KEGG pathway enrichment analysis was performed using MetaboAnalyst 4.0, and dot plots were generated with the ggplot2 package in R software. Heatmaps were generated using the heatmap package in R software.

### Glucose/glutamine consumption assay and targeted metabolic analysis

For glucose/glutamine consumption assay, cells were cultured in medium for 48 h. One milliliter of culture medium was collected to detect the glucose and glutamine in culture medium. For the detection of other metabolites in cells, approximately 1 × 10^7^ cells were collected for targeted metabolic analysis. The culture medium and cells were then mixed with 0.8 mL of chloroform/methanol (2:1, v/v, Thermo Fisher Scientific) and homogenized for 2 min. The mixture was incubated at -20 °C for 15 min and centrifuged at 13000 *g* for 10 min at 4 °C. The upper phase was then evaporated under a nitrogen stream. Metabolites were redissolved in a 100 μL solution of acetonitrile/water (99:1, v/v, Anpel laboratory technologies, Shanghai, China) and detected using an Ultra Performance Liquid Chromatography system (UPLC, Waters Corporation, Milford, MA, USA) coupled with a Quadrupole Time-Of-Flight mass spectrometer (Q-TOF MS, Waters Corporation). Standard metabolites for TCA intermediates, glucose, and glutamine were purchased from Sigma-Aldrich. All data were analyzed with the MassLynx software (Waters Corporation) and normalized with cell numbers.

### Fatty acid staining

Fatty acids were stained with BODIPY ^®^ lipid probes (Thermo Fisher Scientific) according to the manufacturer's instructions. Briefly, 2 × 10^4^ cells were probed with 2 μM lipid indicator for 30 min in a dark room and washed three times with PBS. Photographs were captured by a fluorescence microscope (Nikon Corporation). Quantitative data were obtained in terms of the gray value using Image-Pro Plus 6.0 (Media Cybernetics).

### Glucose and glutamine-based metabolic flux

Cells (5 × 10^5^) were seeded in 60 mm dishes at 37 °C in a 5% CO_2_ incubator 1 day prior to the assay, as previously described 40. For glutamine labeling, cells were cultured with glutamine-free DMEM, and 12% dialyzed calf serum, with the addition of 2 mM ^13^C_5_-Glutamine over 24 h to reach the steady state 41. For glutamine flux assay, cells were cultured in the medium for 0, 30, 60, 120, and 240 min. For glucose labeling, 25 mM ^13^C_5_-Glucose was added in glucose free culture medium supplemented with 12% dialyzed calf serum for 24 h before metabolite extraction. Cells were then washed twice with PBS and sent to Profleader Corporation (Shanghai, China) in dry ice for metabolic flux analysis. Data were collected in full scan mode and normalized via isotope correction analysis.

### Mitochondrial isolation and enzyme activity assay

Mitochondria isolated from cultured cells were used for the detection of complex II enzyme activity 42. The activity of succinate-coenzyme Q reductase (SQR) and succinate dehydrogenase (SDH) was tested as previously described 43. For SQR activity, mitochondria isolated from 2 × 10^7^ cells were incubated with potassium phosphate buffer (KPi buffer) containing succinate (20 mM, Sigma-Aldrich), antimycin (32 μM, Sigma-Aldrich), rotenone (12 μM), NaN_3_ (5 mM, Sangon Biotech), 2,3-dimethoxy-5-methyl-6-geranyl-1,4-benzoquinone (DB, 20 μM, Sigma-Aldrich), and 2,6-Dichlorophenolindophenolsodium salt (DCIP, 50 μM, Sigma-Aldrich). Absorbance was monitored at 600 nm. For SDH activity, mitochondria were incubated with Tris-HCl buffer containing succinate (10 mM), 60 μg/mL methylthiazolyldiphenyl-tetrazolium bromide (MTT, Sigma-Aldrich), 120 μg/mL PMS, antimycin (32 μM), rotenone (12 μM), and NaN_3_ (5 mM). Absorbance was monitored at 570 nm. The data were normalized with the mitochondrial protein concentration.

### Flavin adenine dinucleotide (FAD) assay

A FAD assay was performed using a FAD assay kit (Abcam) according to the manufacturer's instructions. Briefly, 1× 10^6^ cells were lysed with the assay buffer and deproteinated with 50% trichloroacetic acid. FAD in lysate functions as a cofactor in an enzymatic reaction and produces fluorescence with OxiRed probe. Fluorescence (Ex/Em = 535/587 nm) was measured by SpectraMax iD3 Reader (Molecular Devices).

### Gene expression profiling and bioinformatic analysis

Cells were cultured in 60 mm dishes with the input of fresh regular medium for at least 48 h. Approximately 3 × 10^6^ cells were collected and treated with 1 mL ice-cold Trizol reagent (Thermo Fisher Scientific). Cells were then sent to Novogene Corporation on dry ice for gene expression profiling with Novoseq 6000. Data were normalized with a scaling factor using the edgeR program package.

For the public database bioinformatics analysis, RNA-seq data of PDAC samples and pancreatic normal tissue were downloaded from the TCGA and GTEx, respectively. The DESeq2 R package (version 1.24.0) was used for differential expression analysis and gene expression normalization. Gene set enrichment analysis was performed using GSEA software (version 4.0.3) 44. Enriched terms with FDR value below 0.25 were considered statistically significant.

### Glucose uptake assay

A glucose uptake assay was performed as previously reported 45. Briefly, cells were seeded at a density of 2 × 10^6^ cells/well in 6-well plates and then washed twice with PBS. Thereafter, cells were cultured in 500 μL KRH buffer (131 mM NaCl, 5 mM KCl, 1.3 mM MgSO_4_, 1.3 mM CaCl_2_, 0.4 mM KH_2_PO_4_, 6 mM Glucose, 6 mM HEPES, pH 7.4) with 0.1 mM 2-(N-(7-Nitrobenz-2-oxa-1,3-diazol-4-yl) Amino)-2-Deoxyglucose (2-NBDG, Thermo Fisher Scientific) for 30 min at 37 ℃. Cells were then washed twice with KRH buffer. Fluorescence was monitored at an excitation/emission of 485/535 nm using a SpectraMax iD3 Reader (Molecular Devices) and normalized to cell number.

### Cardiolipin content assay

The cardiolipin assay kit (ab241036, Abcam) was used to quantify the cardiolipin according to the manufacturer's instructions. Briefly, for cultured HEK293 cells, cells were trypsin digested and washed with PBS. Cells were then suspended in CL assay buffer and lysed by using sonicator. For mice heart issue, samples were homogenized in CL assay buffer and further lysed by using sonicator. After Centrifuge at 10000 g for 10 min at 4°C, the supernatant was transferred to a fresh tube and the protein concentration of that was quantified. Each sample containing 20 μg protein were mixed with 50 μL probe then incubated at room temperature for 10 min. Finally, fluorescence can be recorded at Ex/Em = 340/480 nm using a SpectraMax iD3 Reader (Molecular Devices).

### Colony formation assay

Cells were seeded at a density of 1 × 10^3^ cells/well in 6-well plates and cultured at 37 °C in a 5% CO_2_ incubator for 14 days. Colonies were fixed with 4% paraformaldehyde and stained with crystal violet (Beyotime Biotechnology) for 20 min at room temperature about 25 ℃. The number of colonies was counted using a Gel-Pro Analyzer 4.0 (Media Cybernetics).

### Statistical analysis

All quantitative experiments were repeated independently at least 3 times. Data are presented as means ± standard error of the mean (SEM). All statistical analyses were performed using SPSS 22.0 (IBM, Armonk, NY, USA). Significance (*P* ≤ 0.05) was calculated using the two-tailed Student's* t* test.

## Results

### COX7A2L regulates complex II-dependent mitochondrial respiration

**Figure 1:**
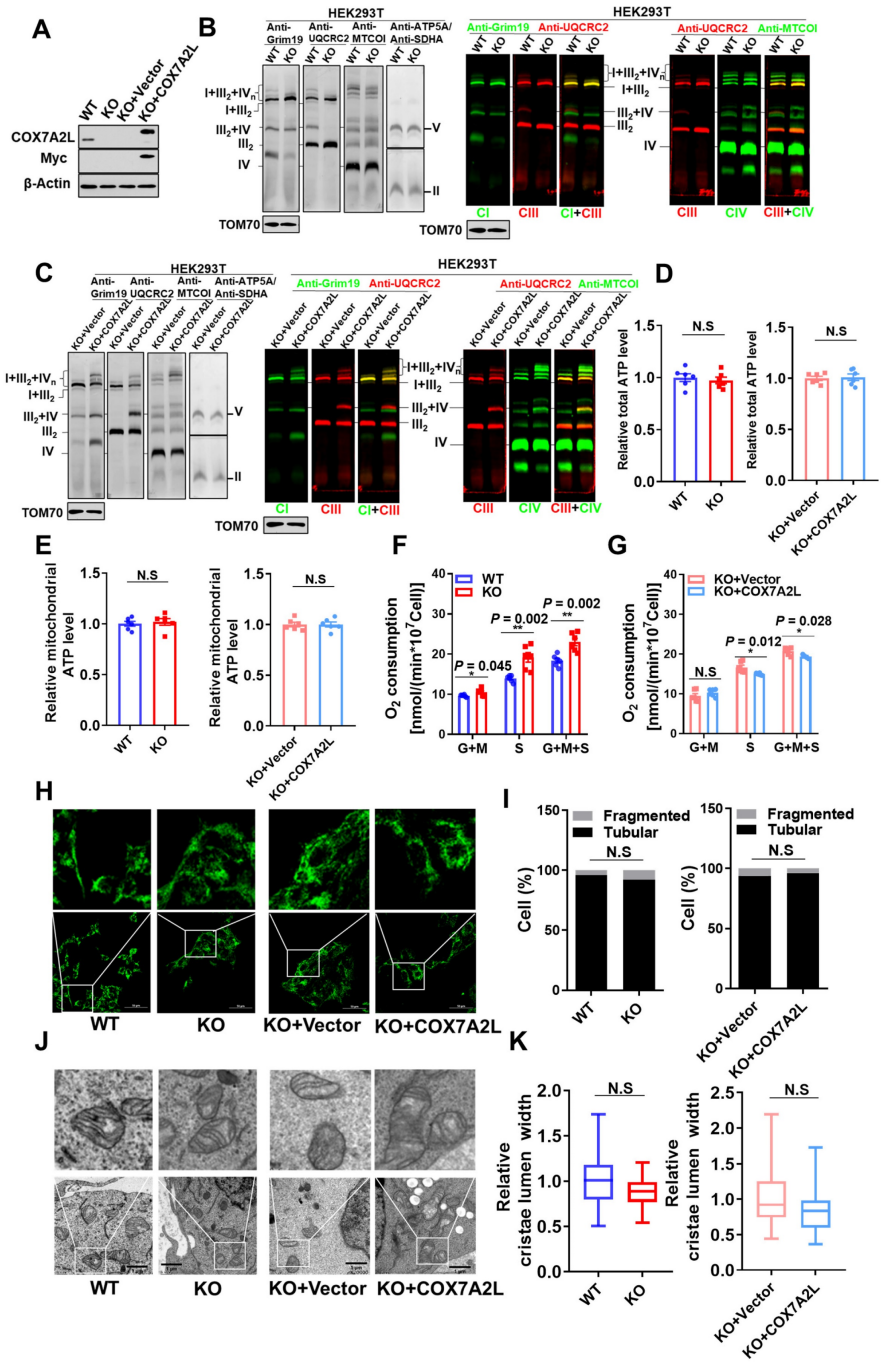
**COX7A2L regulates complex II-dependent mitochondrial respiration.** (**A**) Western blot analysis of COX7A2L and Myc Tag in control HEK293T cells (WT), *COX7A2L^-/-^* cells (KO), and *COX7A2L^-/-^* cells infected with lentivirus containing an empty vector (KO+Vector) or COX7A2L cDNA (KO+COX7A2L). β-Actin was used as an internal control. (**B-C**) Blue native PAGE (BNG)/immunoblotting analysis of respiratory chain supercomplexes in WT and KO (**B**) as well as KO+Vector and KO+COX7A2L (**C**) mitochondria solubilized with digitonin. Complex I-V were immunoblotted with anti-Grim19, anti-SDHA, anti-UQCRC2, anti-MTCOI, and anti-ATP5A antibodies, respectively. Alkaline phosphatase-conjugated or fluorescence- conjugated secondary antibodies were used for differently presenting BNG. Samples were denatured and applied to SDS-PAGE for the incubation of anti-TOM70 as a loading control. (**D-E**) Relative total **(D)** and mitochondrial ATP levels **(E)** in WT, KO, KO+Vector, and KO+COX7A2L cells (n = 6). (**F-G**) Glutamate and malate (G+M)-dependent, succinate (S)-dependent, and glutamate, malate, and succinate (G+M+S)-dependent respiration in WT and KO (**F**) as well as KO+Vector and KO+COX7A2L cells (**G**) with 6 independent replicates. (**H-I**) Immunofluorescence analysis of WT and KO as well as KO+Vector and KO+COX7A2L cells. Mitochondria were stained with anti-TOMM20 antibodies (**H**). Fragmentated and tubular mitochondria were counted (**I**) (100 cells per group). Scale bars, 50 μm. (**J-K**) Transmission electron microscopy of mitochondria in WT and KO as well as KO+Vector and KO+COX7A2L cells (**J**). Average mitochondrial cristae lumen widths (**K**) were determined with 3 cristae per mitochondria, with a total of ten mitochondria each group. Scale bars, 1 μm. Quantitative data are presented as mean ± SEM. N.S, not significant; * *P* ≤ 0.05, ** *P* ≤ 0.01.

To investigate the metabolic contribution of COX7A2L in mammalian cells, we first generated the *COX7A2L*^-/-^ cell model and evaluated the regulatory role of COX7A2L in mitochondrial function. CRISPR/Cas9 technology was used to generate *COX7A2L^-/-^* 293T cells based on a published protocol from Zhang Feng's lab 46 (**Figure 1A**). Whole genome sequencing followed by off-target analysis revealed that the CRISPR/Cas9-based editing procedure in *COX7A2L^-/-^* 293T cells did not cause off-target alterations in the coding regions of other genes (**Supplementary dataset 1**; unavailable for initial submission). Although the use of CRISPR/Cas9 technology is limited by off-target effects owing to the non-specific binding of guide RNA (gRNA) to genomic DNA 47, the overexpression of Myc-tagged wild-type *COX7A2L* in *COX7A2L^-/-^* 293T cells was used as a parallel model to confirm the biological contribution of COX7A2L observed in *COX7A2L^-/-^* 293T cells (**Figure 1A**). KO of *COX7A2L* in 293T cells led to impaired assembly of CIII_2_+IV supercomplex and a respirasome with a higher molecular weight than that of CI_1_+CIII_2_+CIV_1_ (**Figure 1B**), whereas the *COX7A2L^-/-^* cells over-expressing *COX7A2L* restored and upregulated mitochondrial supercomplex assembly (**Figure 1C**), likely because of higher COX7A2L levels in KO+COX7A2L cells than those in wildtype 293T cells (**Figure 1A**). Moreover, Lobo-Jarne, T., et al. previously found that *COX7A2L^-/-^* 293T led to increased complex III assembly 48, which may also contribute to the increase in CIII dimmers and I+III_2_ supercomplexes (**Figure 1B-C**). Altogether, our results agree with a previous study showing that the loss of COX7A2L has little effect on respirasomes containing one copy of CIV 14. However, we found that COX7A2L seems to be required for the assembly of respirasomes containing more than one copy of CIV.

Although neither total nor mitochondrial ATP levels were affected by COX7A2L (**Figure 1D-E**), we found that COX7A2L negatively regulated CII-mediated mitochondrial respiration in 293T cells (**Figure 1F-G**). We believe that the regulatory role of COX7A2L in CII-mediated mitochondrial respiration is not cell type-specific as both COX7A2L-depleted mouse C2C12 and 3T3-L1 cells (**Figure S1A-B**) exhibited a failure of respirasome assembly (**Figure S1C-D**), with CII-mediated respiration being upregulated (**Figure S1E-F**) when compared with that in control cells.

Moreover, the change of CII acitivy and supercomplex assembly in COX7A2L cells is unlikley due the change of cardiolipin (**Figure S1G**). To further analyze the contribution of COX7A2L to endogenous mitochondrial respiration, we analyzed mitochondrial morphology, which is sensitive to changes in mitochondrial respiration and oxidative phosphorylation (OXPHOS) in 293T cells, with or without COX7A2L 49. However, both mitochondrial fragmentation (**Figure 1H-I**) and cristae lumen width (**Figure 1J-K**), as determined by confocal microscopy and transmission electron microscopy, respectively, were not significantly affected by COX7A2L depletion. The gene set enrichment analysis (GSEA) failed to yield mitochondrial respiration and OXPHOS-related pathways based on transcriptional data from two 293T cell models (**Supplementary dataset 2**; false discovery rate (FDR) < 0.25; unavailable for initial submission). Overall, our results indicated that COX7A2L negatively regulates CII-mediated mitochondrial respiration.

### COX7A2L regulates glutamine-dependent cell survival and proliferation

**Figure 2:**
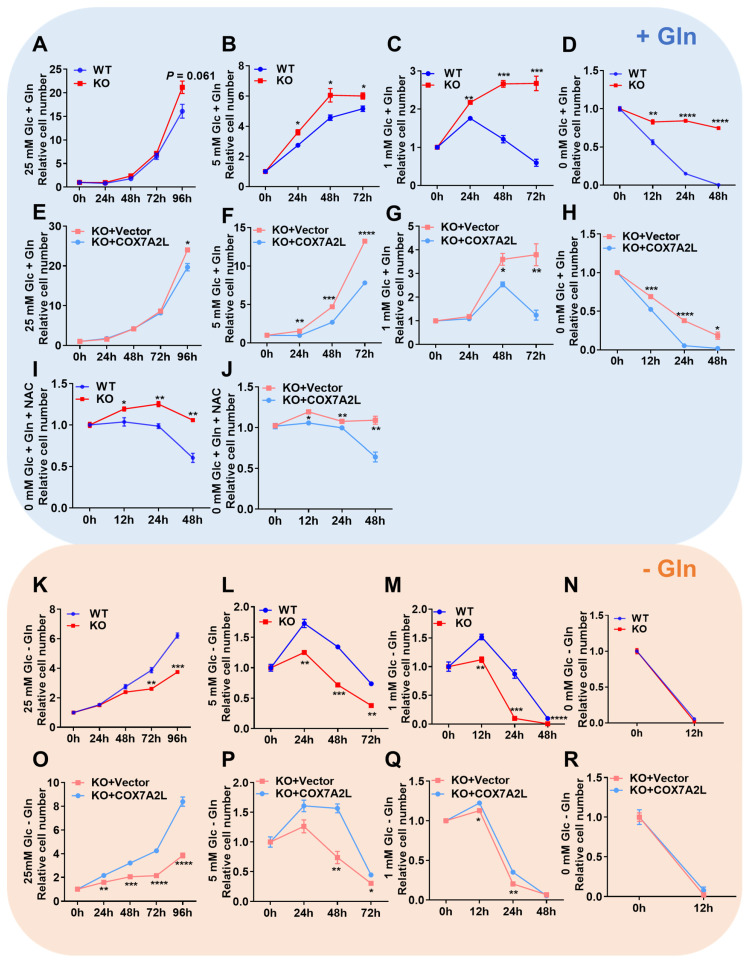
** COX7A2L regulates glutamine-dependent cell survival and proliferation.** (**A-H**) Cell proliferation of WT and KO as well as KO+Vector and KO+COX7A2L cells under culture condition of 25 mM (**A**, **E**), 5 mM (**B, F**), 1 mM (**C, G**) and 0 mM (**D, H**) glucose in the presence of 4 mM glutamine. (**I-J**) Cell proliferation of WT and KO (**I**) as well as KO+Vector and KO+ COX7A2L (**J**) cells in glucose-free medium containing 5 mM NAC, a ROS scavenger. (**K-R**) Cell proliferation of WT and KO as well as KO+Vector and KO+COX7A2L cells under culture condition of 25 mM (**K, O**), 5 mM (**L, P**), 1 mM (**M, Q**) and 0 mM (**N, R**) glucose in the absence of glutamine. Cells were counted at the indicated time point with 3 independent replicates. Relative cell number were normalized to the initial time point (0 h) when cells were first replaced with the corresponding medium. Data are presented as mean ± SEM. N.S, not significant; *P ≤ 0.05, **P ≤ 0.01, ***P ≤ 0.001, ****P ≤ 0.0001.

To assess the cellular effects of COX7A2L on mitochondrial supercomplex assembly, we measured cell proliferation in 293T cells with or without COX7A2L. The growth rate of *COX7A2L*^*-/-*^ 293T cells was significantly higher and *COX7A2L^-/-^* cells over-expressing *COX7A2L* was lower than that of control cells in medium containing 25 mM glucose and 4 mM glutamine (**Figure 2A, E**). We then expressed different COX7A2L forms in 293T cells, including wild-type (WT) and mutant COX7A2L (Y73A, Y-A; depletion of VP, 70DEL_VP) (**Figure S2A**), where mutant forms Y73A and 70DEL_VP specifically impaired protein-protein interactions during supercomplex assembly 24. As shown in **Figure S2B**, only the WT form was able to restore supercomplex assembly. Further, the over expression of the WT form resulted in a significantly lower cell proliferation of *COX7A2L^-/-^* 293T cells when compared with the over expression of Y73A and VP (**Figure S2C**). These results demonstrated that COX7A2L negatively regulated cell proliferation, likely through its effect on supercomplex assembly. Furthermore, we found that *COX7A2L^-/-^* cells were resistant to glucose starvation but *COX7A2L^-/-^* cells over-expressing *COX7A2L* has poor resistance when compared with control cells (**Figure 2B-D**; **Figure 2F-H**). Apoptosis analysis revealed that the loss of COX7A2L did not induce apoptosis when 293T cells were cultured in medium containing considerable amounts of glucose (**Figure S2D-E** for 25 mM glucose; **Figure S2F-G** for 5 mM glucose), suggesting that the increased number of viable *COX7A2L^-/-^* cells was because of enhanced cell proliferation when compared with WT 293T cells. Furthermore, *COX7A2L^-/-^* cells were resistant to glucose deprivation-induced cell apoptosis compared with control cells (**Figure 2D, H; Figure S2H, I**). Although mitochondrial ROS generation in *COX7A2L^-/-^* cells cultured in a high-glucose medium did not differ from that in control cells (**Figure S2J**), *COX7A2L^-/-^* cells cultured in glucose-depleted medium generated less mitochondrial ROS (**Figure S2K**) and H_2_O_2_ (**Figure S2L**). Administration of NAC, a ROS scavenger, narrowed the difference in cell viability (**Figure 2I-J**), particularly in the early 24 h, between *COX7A2L^-/-^* cells and control cells in glucose-depleted medium, probably because of the antioxidant activity of NAC (**Figure S2M-N**). These results indicate that *COX7A2L^-/-^* cells have a higher antioxidant potential than WT 293T cells. However, whether the increase in antioxidant potential is due to the increased levels and activity of antioxidant enzymes and/or redox capacity remains to be further studied. Together, our results revealed that the loss of COX7A2L promoted cell proliferation and protected cells from oxidative damage in a glucose-independent manner.

Glutamine is another major carbon source that can support cell proliferation and protect cells from H_2_O_2_-induced damage. We investigated whether COX7A2L relies on glutamine to regulate cell proliferation. As shown in **Figure 2K-M**, the loss of *COX7A2L* inhibited the cell proliferation of 293T cells cultured in glutamine-deprived medium, whereas the overexpression of endogenous *COX7A2L* in *COX7A2L^-/-^* cells increased cell proliferations compared with that of control *COX7A2L^-/-^* cells (**Figure 2O-Q**). These differences were abolished in a medium lacking both glucose and glutamine (**Figure 2N and R**). To validate this observation, *COX7A2L* was knocked down (KD) in HEK293T cells (**Figure S2O**). KD of *COX7A2L* led to impaired assembly of respirasome with higher molecular weight than that of classic respirasome (I+III_2_+IV) (**Figure S2P**).

Consistent with the observation in COX7A2L^-/-^ cells, KD of COX7A2L could increase CII and CI+CII-dependent respiration compared with that in control cells (**Figure S2Q**). *COX7A2L* KD cells grew faster than control cells in regular medium and were resistant to death in a medium lacking glucose in the presence of glutamine (**Figure S2R-S**). Glutamine removal eliminated both effects (**Figure S2T-U**). These results further support the notion that COX7A2L is essential for respirasome formation and that cells lacking COX7A2L are more sensitive to glutamine deprivation. To determine whether the impairment of respirasome and/or supercomplex CIII_2_+CIV contributes to the nutritional preference in cells lacking COX7A2L, CI-containing supercomplexes, which include respirasome, were depleted by knocking out NDUFB6 in HEK293T cells with or without the KD of COX7A2L (**Figure S2V**). Therefore, only supercomplex CIII_2_+CIV differed between NDUFB6*^-/-^* cells and COX7A2L knockdown NDUFB6*^-/-^* cells (**Figure S2W**). However, supercomplex CIII_2_+CIV depletion neither affected CII-dependent respiration nor changed the cell proliferation rate (**Figure S2X-Y**).

Altogether, our results suggest that COX7A2L-expressing cells prefer glucose, whereas *COX7A2L^-/-^* cells favor glutamine as a metabolic carbon source for cell metabolism. We hypothesize that this nutrient preference is largely mediated by the changes in mitochondrial respirasome assembly.

### Loss of COX7A2L leads to alteration of metabolic features

**Figure 3:**
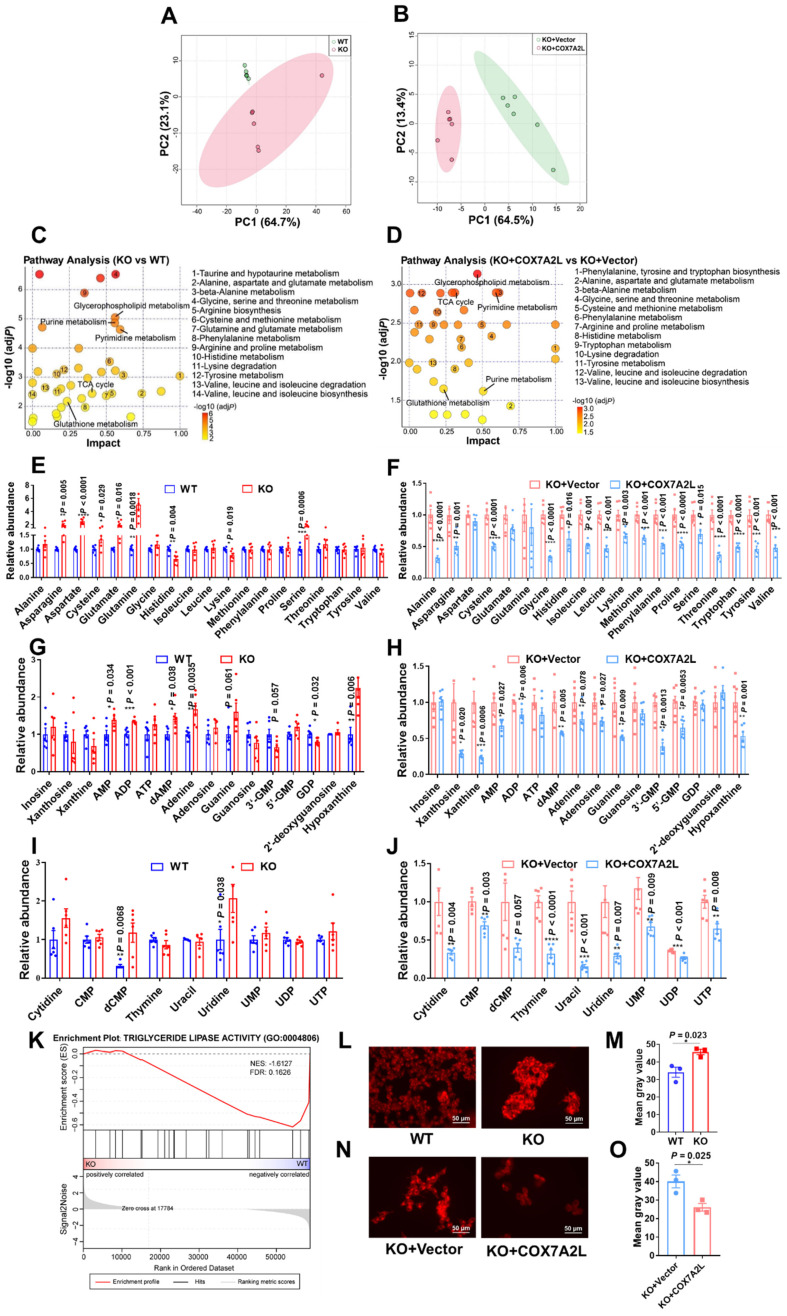
** Loss of COX7A2L leads to alteration of metabolic features.** (**A-B**) Principal component (PC) analysis of WT and KO (**A**) as well as KO+Vector and KO+COX7A2L (**B**) cell samples in untargeted metabolomics study. (**C-D**) Metabolite set enrichment analysis of WT and KO (**C**) as well as KO+Vector and KO+COX7A2L cells (**D**). (**E-J**) Relative amino acid (**E-F**), purine (**G-H**), and pyrimidine (**I-J**) abundances in WT and KO as well as KO+Vector and KO+COX7A2L cells. Data were obtained from untargeted metabolomics (n = 6) and outliers were excluded in the statistical analysis. (**K**) Gene set enrichment analysis of triglyceride lipase activity associated gene set in KO and WT cells. Data were obtained from gene expression profiling. (**L-O**) Fatty acid staining and quantitative analysis in WT and KO (**L-M**) as well as KO+Vector and KO+COX7A2L cells (**N-O**) with 3 independent replicates. Scale bars, 50 μm. Quantitative data are presented as the mean ± SEM. *P ≤ 0.05, **P ≤ 0.01, ***P ≤ 0.001, ****P ≤ 0.0001.

While our results suggest that the nutrient preference of *COX7A2L^-/-^* cells changes from glucose to glutamine during cell proliferation when compared with that of control cells, we believe that cells with and without COX7A2L may have distinct metabolic profiles. To test this hypothesis, the metabolic features of two 293T cell models, with or without COX7A2L, were determined based on an untargeted metabolic profiling strategy. PCA revealed that cells exhibited distinct metabolic profiles depending on COX7A2L competence (**Figure 3A-B**). Metabolite set enrichment analysis highlighted significant differences in amino acid metabolism pathways (14 out of all 40 significantly enriched pathways) between *COX7A2L^-/-^* and control cells (**Figure 3C**, **Supplementary dataset 3**; FDR < 0.25; unavailable for initial submission). Most amino acid pathways were also enriched in *COX7A2L^-/-^* cells when COX7A2L was overexpressed (13 out of all 38 significantly enriched pathways; **Figure 3D**; **Supplementary dataset 3**; FDR < 0.25 unavailable for initial submission). Notably, the regulatory effects of COX7A2L on glutamate/glutamine and antioxidant pathway (**glutathione**) metabolism were also confirmed (**Figure 3C-D**), supporting our hypothesis that *COX7A2L^-/-^* cells preferred glutamine metabolism. Moreover, cells with or without COX7A2L exhibited altered purine and pyrimidine metabolism (**Figure 3C-D**). To further investigate whether these enriched pathways are over-activated or repressed in *COX7A2L^-/-^* cells, we performed relative quantitative analysis of amino acids, purines, and pyrimidines. COX7A2L KO cells exhibited higher and *COX7A2L^-/-^* cells over-expressing *COX7A2L* exhibited lower levels of amino acids (**Figure 3E-F**), purines (**Figure 3G-H**), and pyrimidines (**Figure 3I-J**) when than control cells. Furthermore, increased glycerophospholipid synthetic activity and decreased fatty acid oxidation were observed in *COX7A2L^-/-^* cells. This is attributable to higher levels of glycerophospholipids (**Figure S3**) as confirmed by decreased triglyceride lipase activity (**Figure 3K**) and higher fatty acid content compared with that in matched control cells (**Figure 3L-M** for *COX7A2L^-/-^* cells versus *COX7A2L^+/+^* cells; **Figure 3N-O** for *COX7A2L^-/-^* cells versus *COX7A2L^-/-^* cells over-expressing *COX7A2L*). Taken together, these results suggest that the loss of COX7A2L alters cell metabolic features.

### Phenotypic alteration and preference of carbon source in *Cox7a2l^-/-^* mice

**Figure 4:**
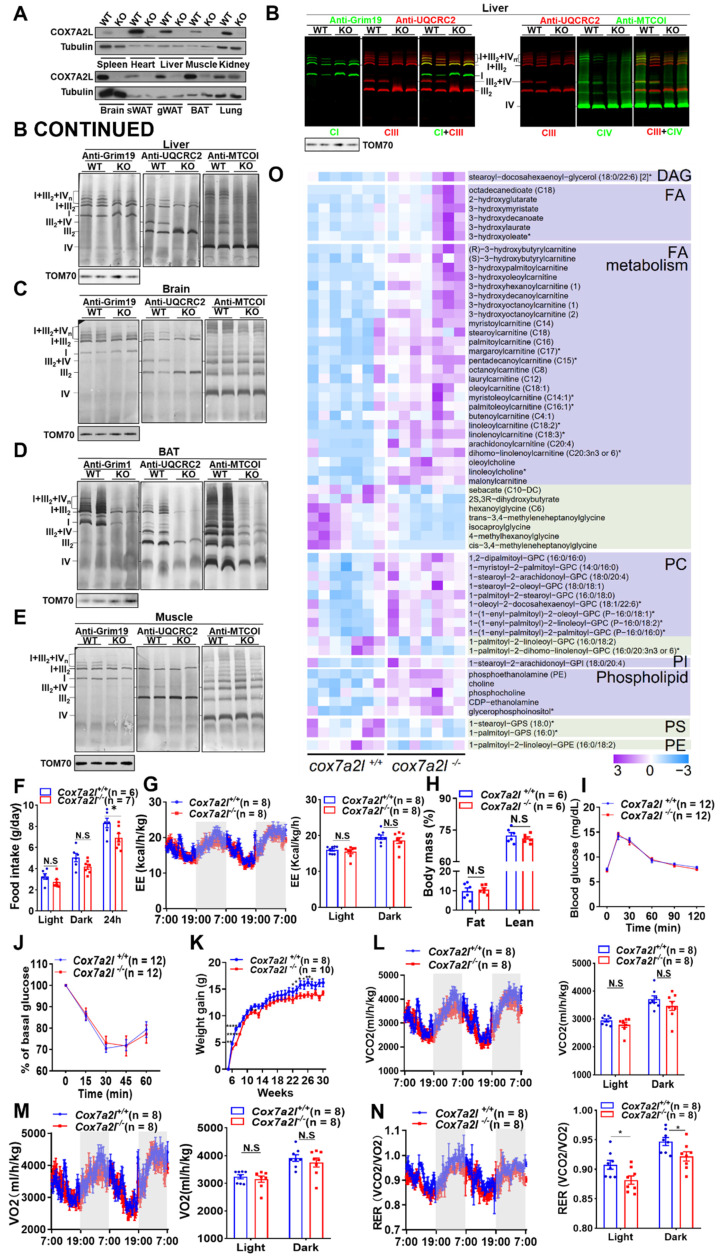
** Phenotypic alteration and preference of carbon source in *Cox7a2l^-/-^* mice.** (**A**) WB analysis of COX7A2L in 10 indicated tissues from DBA/2J mice with (WT) or without Cox7a2l (KO). sWAT and gWAT, subcutaneous and gonadal white adipose tissue; BAT, brown adipose tissue. Tubulin was used as an internal control. (**B-E**) Blue native PAGE/immunoblotting analysis of respiratory chain supercomplexes in liver (**B**), brain (**C**), BAT (**D**) and muscle (**E**) tissue from WT and KO mice. Complexes I, III, and IV were immunoblotted with anti-Grim19, anti-UQCRC2, and anti-MTCOI antibodies, respectively. Samples were denatured and applied to SDS-PAGE for the incubation of anti-TOM70 as a loading control. (**F**) Daily food intake of *Cox7a2l^+/+^* and *Cox7a2l^-/-^* mice. (**G**) Energy expenditure analysis of *Cox7a2l^+/+^* and *Cox7a2l^-/-^* mice. (**H**) Body mass (fat mass and lean mass) of *Cox7a2l^+/+^* and *Cox7a2l^-/-^* mice (26 weeks old). (**I-J**) Glucose tolerance test (GTT, **I**) and insulin tolerance test (ITT, **J**) of *Cox7a2l^+/+^* and *Cox7a2l^-/-^* mice. (**K**) Gain of body weight of *Cox7a2l^+/+^* and *Cox7a2l^-/-^* mice. **(L-M)** Carbon dioxide production (VCO_2_, **L**) and oxygen consumption (VO_2_, **M)** of *Cox7a2l^+/+^* and *Cox7a2l^-/-^* mice. (**N**) Respiratory exchange ratio (RER) of *Cox7a2l^+/+^* and *Cox7a2l^-/-^* mice. (**O**) Heat map of fatty acid metabolites in *Cox7a2l^+/+^* and *Cox7a2l^-/-^* mice. Data were obtained from untargeted metabolomics of mouse quadriceps femoris (n = 7). Quantitative data were normalized with wildtype mice and presented as mean ± SEM. N.S, not significant; * *P* ≤ 0.05, **P ≤ 0.01, ****P ≤ 0.0001.

In contrast to human COX7A2L, which contains 113 amino acids, two forms of *cox7a2l* with 111 (short form) or 113 (long form) amino acids were found in different mouse strains 23. While the short form is non-functional, we generated *Cox7a2l^-/-^* mice using the DBA/2J mouse strain with long form COX7A2L (**Figure 4A**). KO of *cox7a2l* led to impaired assembly of CIII_2_+IV supercomplex and respirasomes CI_1_+CIII_2_+CIV_n_ in liver, brain, brown fat, and muscle (**Figure 4B-E**). Mitochondrial complexes underwent an overall dramatic decrease in brown fat because of COX7A2L depletion (**Figure 4D**), indicating that COX7A2L is physiologically important for the assembly of respirasome. The cardiolipin level was not affected by depletion of COX7A2L in the mouse liver as we found in HEK293T cells (**Figure S4A**), which further exclude the contribution of cardiolipin in COX7A2L depletion mediated respiratory chain complex remodling. Although *Cox7a2l^-/-^* mice showed decreased food intake (**Figure 4F**), energy expenditure was consistent between *Cox7a2l^-/-^* and* Cox7a2l^+/+^*mice (**Figure 4G**). Moreover, the composition of lean and fat masses (**Figure 4H**), glucose homeostasis (**Figure 4I**), and insulin sensitivity (**Figure 4J**) were not affected by COX7A2L. These data suggest that decreased food intake in *Cox7a2l^-/-^* mice may contribute to lower weight gain in *Cox7a2l^-/-^* compared with that in *Cox7a2l^+/+^*mice (**Figure 4K**). Although carbon dioxide production (**Figure 4L**) and oxygen consumption (**Figure 4M**) were not different between *Cox7a2l^-/-^* and *Cox7a2l^+/+^*mice, a mild decrease in respiratory exchange ratio (RER) was observed in *Cox7a2l^-/-^* mice compared with that in *Cox7a2l^+/+^*mice (**Figure 4N**). It is likely that *Cox7a2l^-/-^* mice and *Cox7a2l^+/+^*mice favored different carbon sources for oxidation. Glucose, fats, and proteins are known carbon sources with RER of 1, ~0.7, and ~0.8, respectively 50, suggesting that *Cox7a2l^-/-^* mice may favor fat and/or protein oxidation when compared with *Cox7a2l^+/+^*mice. Given the fact that (1) glucose homeostasis (**Figure 4I**) and whole-body fats were not significantly affected (**Figure 4H**) by *Cox7a2l* and that (2) fat oxidation is likely to be repressed in mouse muscle, one of the major energy expenditure tissues, since serum free lipids and free fatty acids accumulated in mice skeleton muscle with KO of *Cox7a2l* (**Figure 4O**), we hypothesized that *Cox7a2l^-/-^* mice have increased protein oxidation compared with that in control mice, which agree with the results of a study on zebrafish 26. While the entrance of glutamine into the TCA cycle is the key to the initiation of amino acid oxidation, our data are consistent with our hypothesis that *COX7A2L^-/-^* cells preferred glutamine metabolism.

To test whether lacking *Cox7a2l^-/-^* mice have altered glutamine preference,* Cox7a2l^-/-^* and *Cox7a2l^+/+^*mice were fed with or without glutamine supplementation for 4-5 months. We confirmed that *Cox7a2l^-/-^* mice with standard diet gained less weight than *Cox7a2l^+/+^* mice (**Figure S4B**). However, *Cox7a2l^-/-^* mice with decreased food intake under glutamine supplementation showed a comparable gain in weight as control mice (**Figure S4C-D**). In addition, glutamine supplementation did not change the difference in energy expenditure, carbon dioxide production, oxygen consumption and RER in mice with a standard diet (**Figure S4E-H**). While *Cox7a2l^-/-^* mice ate less, expended the same level of energy, but gained the same level of body weight as control mice during glutamine supplementation (**Figure S4D**), it is likely that *Cox7a2l^-/-^* mice had a better glutamine utilization ability than control mice.

Together, our results suggest that COX7A2L is essential for respirasome assembly and that the loss of COX7A2L may lead to an altered nutrient preference in mice.

### COX7A2L limits the entrance of glutamine-derived carbon source products into the TCA cycle

**Figure 5:**
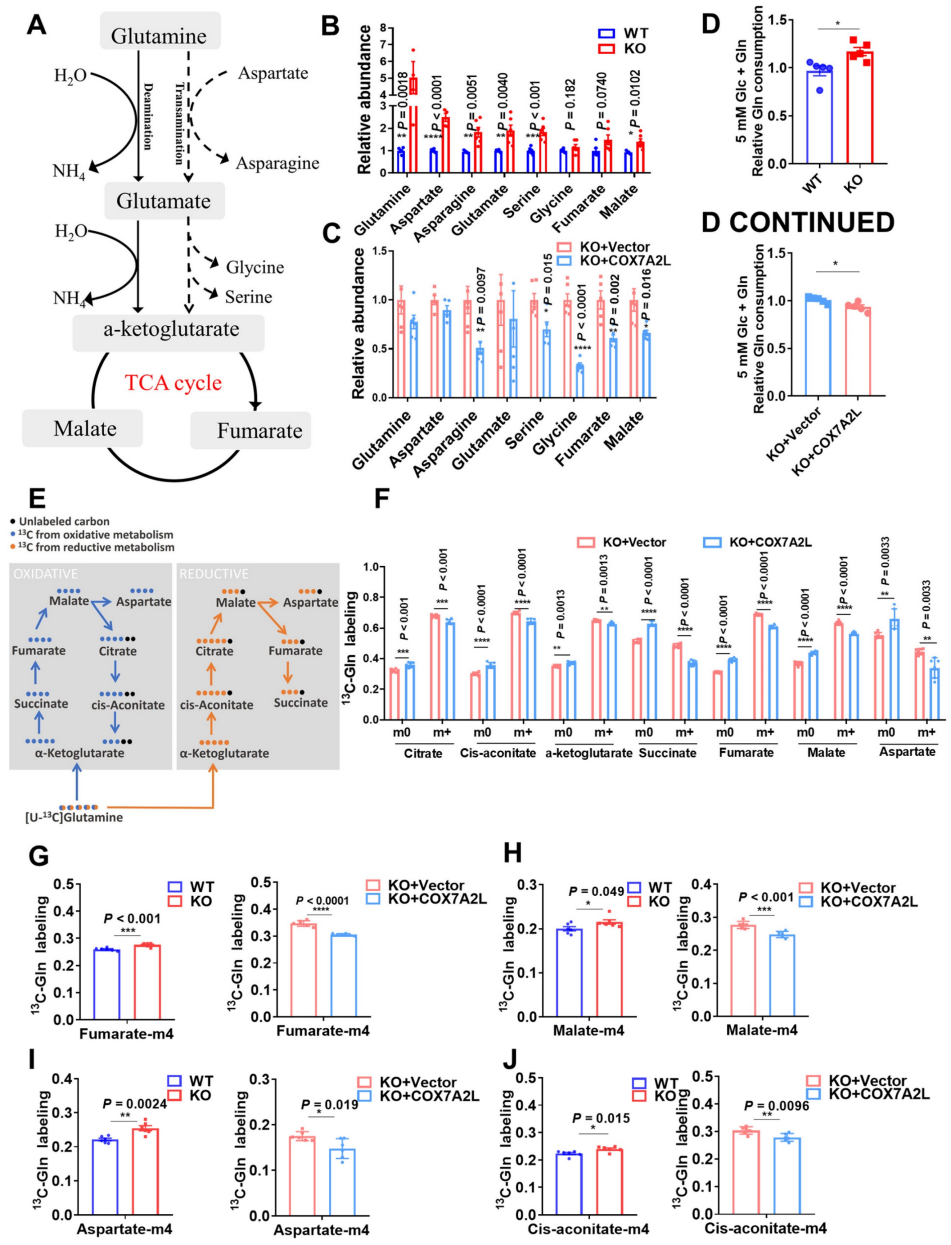
** COX7A2L limits the entrance of glutamine-derived carbon source products into the TCA cycle.** (**A**) Schematic diagram of glutaminolysis. (**B-C**) Relative abundance of metabolites from glutaminolysis pathway in WT and KO (**B**) as well as KO+Vector and KO+COX7A2L (**C**) cells. Data were obtained from untargeted metabolomics (n = 6) and outliers were excluded from statistical analysis. (**D**) Relative glutamine consumption in WT and KO as well as KO+Vector and KO+COX7A2L cells cultured in 5 mM glucose medium (n = 5). (**E**) Schematic diagram of ^13^C_5_-glutamine metabolite labeling pattern. Blue and orange dots indicate ^13^C from oxidative and reductive metabolism, respectively. (**F**) ^13^C_5_-glutamine metabolic flux analysis (MFA) of TCA intermediates in KO+Vector and KO+COX7A2L cells. (**G-J**) ^13^C_5_-glutamine MFA of fumarate (**G**), malate (**H**), aspartate (**I**), and cis-aconitate (**J**) in WT and KO as well as KO+Vector and KO+COX7A2L cells. ^13^C_5_-glutamine metabolic flux analysis was performed in six independent replicates. Quantitative data are presented as mean ± SEM. m0, unlabeled metabolite; m+, labeled metabolite; N.S, not significant; *P ≤ 0.05, **P ≤ 0.01, ***P ≤ 0.001, ****P ≤ 0.0001.

To test the effect of COX7A2L on glucose metabolism, we determined the level of glucose uptake (**Figure S5A**), glucose consumption (**Figure S5B**), and lactate generation (**Figure S5C**) in KO cells and cells expressing COX7A2L. No significant differences were detected. Moreover, in cells cultured with uniformly labeled ^13^C-glucose, the percentages of labeled and unlabeled glycolysis products (pyruvate and lactate) and the pentose phosphate pathway metabolites (Ru5P and R5P) were not affected by COX7A2L (**Figure S5D**), suggesting that COX7A2L does not regulate glucose oxidation for cell proliferation. However, the regulatory effect of COX7A2L on glutamine metabolism was confirmed as *COX7A2L^-/-^* cells had higher levels of TCA intermediate succinate than control cells in mediums that contained glutamine. This difference was not observed in culture medium lacking glutamine (**Figure S5E**). This prompted us to ask whether the loss of *COX7A2L* promotes glutaminolysis to support the biosynthetic features of 293T cells. Untargeted metabolic profiling revealed that metabolic intermediates related to glutaminolysis were upregulated in *COX7A2L^-/-^* cells (**Figure 5A-B**) and downregulated with the over expression of *COX7A2L* (**Figure 5A, C**), indicating that glutaminolysis may be affected by *COX7A2L* depletion. This conclusion was supported by the facts that: (1) *COX7A2L^-/-^* cells exhibited higher levels of glutamine consumption in culture medium (**Figure 5D**); and (2) *COX7A2L^-/-^* cells had higher percentages of labeled glutaminolysis intermediates**,** such as alpha-ketoglutarate, succinate, fumarate malate, citrate, cis-aconitate, and aspartate (**Figure 5E-F**). Moreover, the glutamine tracing kinetic flux assay revealed an increase in glutaminolysis flux via the TCA cycle in *COX7A2L^-/-^* cells when compared with that in cells with overexpressing *COX7A2L* (**Figure S5F-K**). These results indicate that COX7A2L negatively regulates glutaminolysis in mammalian cells. As a major function of glutaminolysis is to supply metabolic intermediates for the TCA cycle 51 (**Figure 5E**), we examined the contribution of glutamine to the TCA cycle. Our results revealed that the percentages of seven unlabeled TCA intermediates increased in *COX7A2L^-/-^* cells over-expressing *COX7A2L* when compared with corresponding control cells (**Figure 5F**). Furthermore, the loss of *COX7A2L* promoted the oxidative metabolism of glutamine in the TCA cycle, while the percentages of TCA intermediates, including fumarate (M+4;** Figure 5E, G**), malate (M+4; **Figure 5E, H**), aspartate (M+4; **Figure 5E, I**), and aconitate (M+4; **Figure 5E, J**) were upregulated in *COX7A2L^-/-^* cells and and downregulated in cells with over-expressing *COX7A2L*, when compared with corresponding control cells.

### COX7A2L regulates glutamine metabolism via respiratory complex II

**Figure 6:**
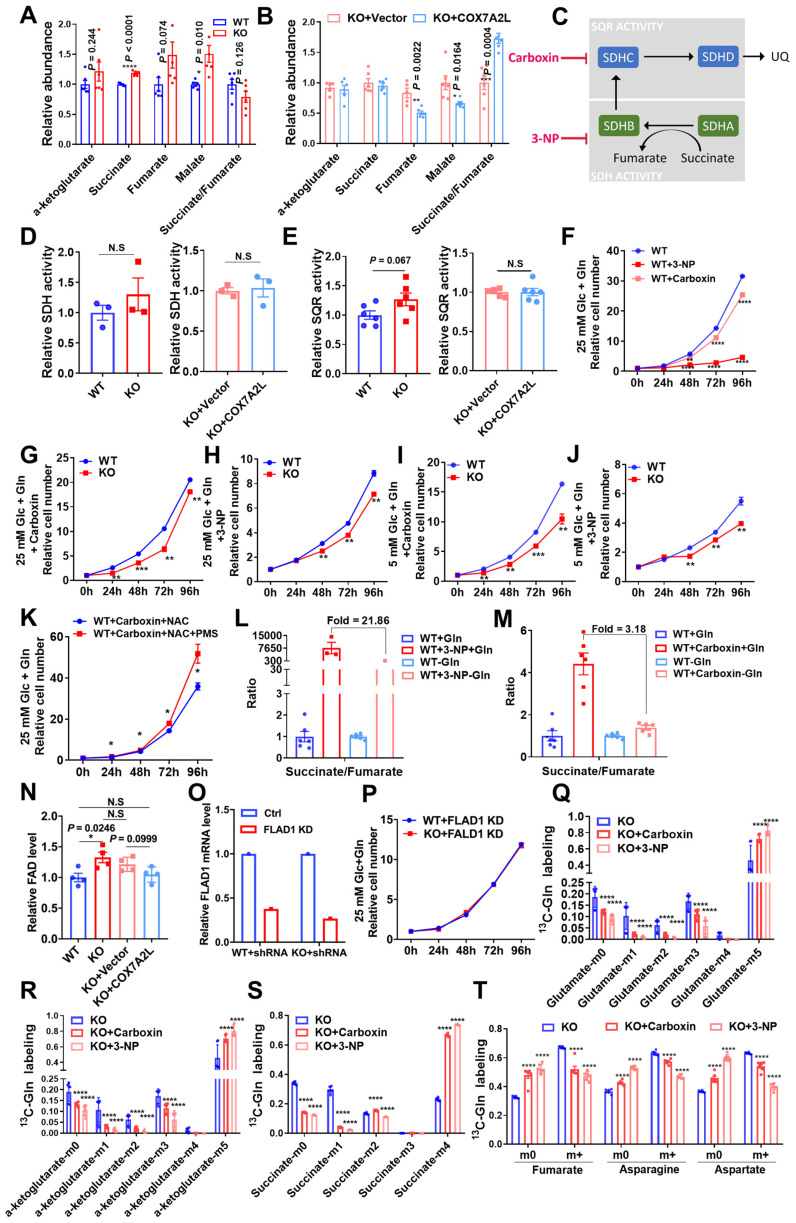
** COX7A2L regulates glutamine metabolism via respiratory complex II.** (**A-B**) Relative abundance of TCA intermediates in WT and KO (**A**) as well as KO+Vector and KO+COX7A2L cells (**B**). Data were obtained from untargeted metabolomics (n = 6), and outliers were excluded from the statistical analysis. (**C**) Schematic diagram of complex II function. Carboxin, a succinate-coenzyme Q reductase (SQR) inhibitor; 3-NP, a succinate dehydrogenase (SDH) inhibitor. (**D-E**) Relative SDH (**D**) and SQR (**E**) activities of mitochondria extracted from WT and KO as well as KO+Vector and KO+COX7A2L cells (n = 3, 6). (**F**) Cell proliferation of WT cells cultured in regular medium with 2 mM 3-NP or 50 μM carboxin. (**G-H**) Cell proliferation of WT and KO cells cultured in regular medium with 50 μM carboxin (**G**) or 2 mM 3-NP (**H**). (**I-J**) Cell proliferation of WT and KO cells cultured in 5 mM glucose medium with 50 μM carboxin(**I**) or 2 mM 3-NP (**J**). (**K**) Cell proliferation of WT cells cultured in regular medium containing both 5 mM NAC and 50 μM carboxin with or without PMS. (**L-M**) The ratio of succinate to fumarate in 293T cells cultured in medium containing or not containing 2 mM 3-NP (**L**) or 50 μM carboxin (**M**) in the presence or absence of glutamine. The data were obtained from targeted metabolic analysis (n = 6). In WT+3-NP+Gln and WT+3-NP-Gln groups, missing samples were due to the undetectable fumarate level in some samples. (**N**) Relative FAD level in 293T cells with differentially expressed COX7A2L. (**O-P**) qPCR validation of FLAD1 knock down in cultured 29T cells (**O**). Cell proliferation with differentially expressed COX7A2L were measured for 96 h **(P)**. (**Q-T**) ^13^C_5_-glutamine metabolic flux analysis of differentialy labeled glutamine (**Q**), alpha-ketoglutarate (**R**), and succinate (**S**) in KO cells after culturing in ^13^C_5_-glutamine labeling medium for 18 h with or without carboxin or 3-NP (**T**). ^13^C_5_-glutamine metabolic flux analysis of glutaminolysis intermediates (fumarate, asparagine, and aspartate) in KO cells after culturing in ^13^C_5_-glutamine labeling medium for 18 h with or without carboxin or 3-NP. Cells were counted at the indicated time point with 3 independent replicates. Relative cell number was normalized to the initial time point (0 h) when cells were first replaced with the corresponding medium. ^13^C_5_-glutamine metabolic flux analysis was performed on six independent replicates. Quantitative data are represented as mean ± SEM. m0, unlabeled metabolites; m+, labeled metabolites. N.S, not significant; **P ≤ 0.01, ***P ≤ 0.001, ****P ≤ 0.0001.

As a supercomplex assembly factor, COX7A2L promotes CI-, CIII-, and CIV-containing supercomplex assembly, which in turn limits complex II-dependent respiration, while CIII and CIV are trapped by CI in the respirasome for CI-dependent respiration 23. In agreement with this notion, 293T cells lacking COX7A2L exhibited higher fumarate levels and a trend toward lower succinate/fumarate ratio (**Figure 6A**), whereas *COX7A2L^-/-^* cells over expressing *COX7A2L* had lower levels of fumarate and a significantly higher succinate/fumarate ratio than corresponding control cells (**Figure 6B**). This indicates that *COX7A2L^-/-^* cells may have higher CII activity than control cells. To further investigate whether SQR, SDH of CII (**Figure 6C**), or electron flux from CII to CIII was responsible for the higher succinate/fumarate ratio in *COX7A2L^+/+^* cells compared with that in *COX7A2L^-/-^* cells, we assessed the SQR and SDH activities and found that both were not affected by the depletion of *COX7A2L* (**Figure 6D-E**). While CII-dependent respiration was higher in *COX7A2L^-/-^*cells and lower in *COX7A2L^-/-^*cells over-expressing COX7A2L (**Figure 1F-G**; **Figure S1E-F**), our results suggested that COX7A2L acts as a negative regulator of CII-mediated respiration by limiting the electron flux from CII to CIII without affecting SQR and SDH activity.

While there is no direct method to inhibit electron transfer from CII to CIII to mimic the mechanism observed in *COX7A2L^-/-^* cells, we used 3-NP and carboxin, which inhibit electron generation from CII via the suppression of SDH and SQR activity, respectively. The inhibition of CII activity owing to SDH or SQR inhibitors effectively reduced cell proliferation in 293T cells (**Figure 6F**). Notably, the inhibition of CII could also reverse the enhanced cell proliferation of *COX7A2L^-/-^* cells when compared with that of control WT cells (**Figure 6G-J**), which further confirmed that CII supports rapid cell proliferation in *COX7A2L^-/-^* cells. In addition, restoring electron transfer in SQR-inhibited cells by adding an electron acceptor of CII (PMS), which accepts and transfersthe electron from the SDH module, could partially promote cell proliferation (**Figure 6K**). We speculate that the CII-driven TCA cycle contributes to cell proliferation. Consistent with our observations in *COX7A2L^+/+^* and *COX7A2L^-/-^* cells, the inhibition of CII by 3-NP or carboxin significantly increased the ratio of succinate/fumarate, whereas the depletion of glutamine dramatically decreased the ratio when compared with that of control cells (**Figure 6L-M**). Moreover, the cellular level of flavin adenine dinucleotide (FAD), a key coenzyme for the oxidation of succinate to fumarate conversion, was negatively associated with the expression of *COX7A2L^-/-^* (**Figure 6N**). This highlighted a quick turnover between FADH_2_ and FAD because of increased CII respiration that facilitated the succinate to fumarate conversion, further accelerating the cell proliferation. This speculation was further confirmed by deleting the FAD synthase, FLAD1, which eliminates the difference between *COX7A2L^+/+^* and *COX7A2L^-/-^* cells in cell proliferation (**Figure 6O-P**). Overall, this suggests that glutamine contributed to the generation of fumarate from succinate, a reaction catalyzed by CII. In cells cultured with uniformly labeled ^13^C-glutamine, inhibition of CII with either 3-NP or carboxin led to the accumulation of glutamate (M+5; **Figure 6Q**), alpha-ketoglutarate (M+5; **Figure 6R**), and succinate (M+4; **Figure 6S**). These were directly produced from glutamine (M+5). Further oxidation of alpha-ketoglutarate (M+5) and succinate (M+4) was repressed by CII inhibition. The percentages of alpha-ketoglutarate (M+1 to M+3) and succinate (M+1 to M+2) were significantly lower in CII-inhibited cells than those in control cells (**Figure 6R-S**). Notably, the over-activation of the reductive carboxylation of glutamine was not observed in CII-inhibited cells, while the proportions of alpha-ketoglutarate (M+4) and succinate (M+3) were similar between cells with active or inhibited of CII (**Figure 6R-S**). Moreover, isotope-labeled fumarate, aspartate, and asparagine were all significantly lower in CII-inhibited cells than those in control cells (**Figure 6T**), further indicating that the entrance of glutamine into the TCA cycle was blocked by CII inhibition.

### Glutamine availability regulates the effect of COX7A2L on the growth of PDAC cells

**Figure 7:**
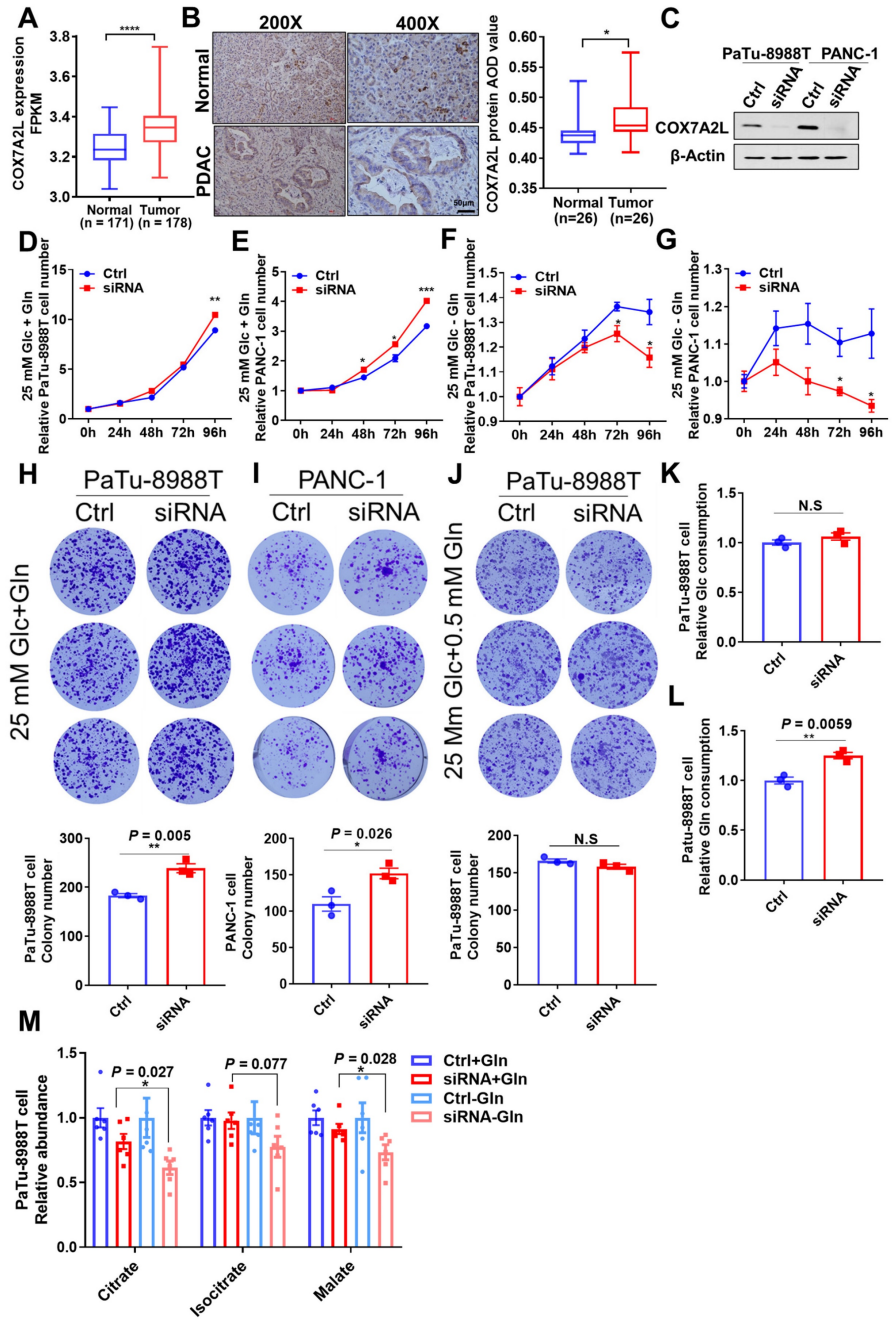
Glutamine availability regulates the effect of COX7A2L on the growth of PDAC cells. (**A**) COX7A2L expression level of normal (from GTEx database, n = 171) and PDAC tissues (from TCGA database, n = 178). (**B**) Immunohistochemical analysis of 26 pancreatic ductal adenocarcinoma (PDAC) specimens and their paired adjacent normal pancreatic tissues performed with anti-COX7A2L antibody. Scale bars, 50 μm. (**C**) Western blot analysis of COX7A2L in PaTu-8988T and PANC-1 cells treated with Ctrl or COX7A2L siRNA. β-Actin was used as an internal control. (**D-G**) Cell proliferation of PaTu-8988T (**D, F**) and PANC- 1 cells (**E, G**) treated with Ctrl or COX7A2L siRNA. Cells were cultured in the presence (**D-E**) or absence (**F-G**) of glutamine. Cells were counted every 24 h with 3 independent replicates. Relative cell number were normalized to that at the initial time point (0 h) when cells were first replaced with the corresponding medium. (**H-J**) Colony formation assay of PaTu-8988T and PANC-1 treated with Ctrl or COX7A2L siRNA. Cells were cultured in 25 mM glucose and 4 mM (**H-I**) or 0.5 mM (**J**) glutamine-containing medium. (**K-L**) Relative glucose consumption (**K**) and glutamine consumption (**L**) in PaTu-8988T cells treated with Ctrl or COX7A2L siRNA (n = 3). (**M**) Relative citrate, isocitrate, and malate abundance of PaTu-8988T cell treated with Ctrl or COX7A2L siRNA. Data were obtained from targeted metabolic analysis (n = 6). Quantitative data are presented as mean ± SEM. N.S, not significant; *P ≤ 0.05, **P ≤ 0.01, ***P ≤ 0.001, ****P ≤ 0.0001.

Most pancreatic ductal adenocarcinoma (PDAC) cells have reprogrammed metabolic pathways with increased glutamine metabolism 52, 53. However, poor nutrient availability in a tumor microenvironment (TME) limits PDAC cell proliferation 54. Specifically, glutamine concentration is significantly lower in the core than that in the peripheral region of the tumor 55. Given the role of COX7A2L in the regulation of glutamine metabolism, it is of great interest to assess how COX7A2L regulates the growth of PDAC cells. The bioinformatic analysis of RNA sequencing data of human PDAC tissues from The Cancer Genome Atlas (TCGA) has revealed the mRNA level of COX7A2L in PDAC is significantly higher than that in normal pancreatic tissue (**Figure 7A**).

Consistently, immunohistochemistry showed that the COX7A2L level was significantly higher in human PDAC tissue than that in the adjacent normal pancreatic tissue (**Figure 7B**). Moreover, in two PDAC cell lines (Patu-8988T and PANC-1), KD of COX7A2L (**Figure 7C**) significantly increased the cell proliferation rate in glucose- and glutamine-containing medium (**Figure 7D-E**) when compared that for control cells. Further, PDAC cells with decreased COX7A2L levels were sensitive to glutamine deprivation (**Figure 7F-G**). These results suggest that COX7A2L may promote PDAC proliferation under nutritional stress, whereas the deletion of COX7A2L may promote PDAC proliferation under unlimited nutrition. Furthermore, although an *in vitro* tumor formation assay in Patu-89088T and PANC-1 cells confirmed that tumorigenesis was enhanced in COX7A2L-deficient cells cultured in glutamine-containing medium (**Figure 7H-I**) in comparison with that in glutamine-depleted medium (**Figure 7J**), an *in vivo* tumorigenesis assay, which generated nutrient-poor microenvironment during tumor growth, showed that COX7A2L expression promoted the growth of PDAC cells when compared with COX7A2L KD (**Figure S6A-E**). Consistently, faster PDAC cell growth was observed in mice overexpressing COX7A2L but not in control mice (**Figure S6F-J**). Metabolic analysis further revealed that glutamine, rather than glucose, consumption was upregulated in 8988T with KD of COX7A2L compared with that in control cells (**Figure 7K-L**). Specifically, the level of TCA intermediates was more sensitive to glutamine deprivation in COX7A2L KD 8988T cells than that in control cells (**Figure 7M**). Altogether, these results suggest that glutamine availability regulates the effect of COX7A2L on the growth of PDAC cells.

## Discussion

Although a supercomplex disruption strategy was previously developed to study the function of supercomplexes in yeast 15, investigating the functional and physiological relevance of the mitochondrial supercomplexes in mammalian cells remains difficult, and discrepancies regarding their function are yet to be resolved 10, 13; this is probably attributable to both the accumulation and depletion of supercomplexes, both of which are accompanied by changes in the function of other respiratory chain complexes. In 2013, COX7A2L was discovered as a mitochondrial supercomplex assembly factor associated with the co-assembly of CI, CIII, and CIV 23, 24. The assembly of individual respiratory chain complexes, however, was not affected by COX7A2L 48. In the present study, we employed the COX7A2L KO model to confirmed that mitochondrial supercomplexes may determine electron flux in the electron transport chain. We further observed that the assembly of mitochondrial CI-, CIII-, and CIV-containing supercomplexes limited CII-dependent respiration without affecting CII activity 23. We also found that mitochondrial supercomplexes controlled the metabolic features of mammalian cells and determined cell fate by dictating whether cells were eligible for proliferation. Importantly, we proposed a regulatory mechanism via which mitochondrial supercomplexes regulate glutaminolysis.

Suppressing succinate oxidation by inhibiting CII activity could prevent the entrance of glutamine into the TCA cycle 56, 57. Glutamine oxidation was downregulated by LND (Lonidamine), a CII inhibitor (*FIGURE 7. LND and TTFA reduce oxidative glutamine metabolism in DB-1 cells*) 58. In addition, increased glutaminolysis in cells with depleted mtDNA may be attributed to the increase in CII function 59, 60. All these findings indicated that the upregulation of CII-related respiration promotes glutamine usage, where an increase in succinate to fumarate conversion may play a central role. In **Figure 6** of our manuscript, we found that the loss of COX7A2L led to a decreased succinate/fumarate ratio; the level of fumarate was significantly higher in *COX7A2L^-/-^* than that in control cells. Largely because COX7A2L ablation blocks the assembly of the respirasome and frees the CIII and CIV for CII-mediated respiration/electron transfer flux 23, thereby promoting the CII-mediated TCA cycling. Moreover, our results on the succinate/fumarate ratio are supported by those of another study 23. Notably, as increased glutaminolysis may fine-tune the nitrogen metabolism of glutamine 61, glutamine-mediated nitrogen metabolism may also contribute to cell proliferation in cells lacking COX7A2L. Overall, we found that increased CII-dependent respiration because of the inhibition of supercomplex assembly by COX7A2L depletion drove nutrient preference for glutamine and promoted cell proliferation. This newly described finding fills a critical knowledge gap regarding the role of mitochondrial supercomplexes in the regulation of metabolism under different physiological conditions 14, 23-25, 48.

COX7A2L is currently the only known factor associated with the assembly of CI, CIII, and CIV. However, its role in supercomplex assembly and mitochondrial respiration has been disputed 14, 23-25, 48. Most COX7A2L-related studies have been carried out in mice with different strains expressing two forms of COX7A2L. The long form (113 amino acids) is functional, whereas the short form (111 amino acids) is not 23. In mouse cells, functional COX7A2L has been found to be essential for the assembly of all CI-, CIII-, and CIV-containing supercomplexes. In the current study, we further explored the role of COX7A2L in supercomplexes and confirmed that the assembly of the mitochondrial respirasomes was mediated by COX7A2L in both human cells and mouse tissues 14. Although COX7A2L was previously shown to promote the assembly of mitochondrial megacomplex CI_2_+CIII_2_+CIV_2_ 48, we did not observe such an alteration where a megacomplex was not detected in our BNG system, possibly because megacomplexes were not able to enter the gel 62. Moreover, our results agreed with Esther Lapuente-Brun et al. regarding the role of COX7A2L in shifting the electron flux from CII to CI, further limiting CII-dependent mitochondrial respiration without affecting endogenous mitochondrial respiration 23. CII is the only respiratory complex that does not co-assemble with other respiratory complexes. Theoretically, the more CIII and CIV assemble into the CI-, CIII-, and CIV-containing supercomplexes, the less they can serve as electron acceptors for CII 23. We observed that both SDH and SQR activities were not affected by COX7A2L depletion. However, CII-dependent respiration was upregulated in *COX7A2L^-/-^* cells, suggesting that excess free CIII and CIV in *COX7A2L^-/-^* cells could be effectively used as electron acceptors of CII outflux. In agreement with a previous 293T cell-based study on COX7A2L published in 2018 48, we observed that OXPHOS function and CII activity were largely unaffected by COX7A2L depletion. However, since succinate-G3P-mediated respiration rather than succinate-mediated respiration was measured in the above-mentioned study 48, it is difficult to discuss whether CII-mediated mitochondrial respiration was affected in their model. Multiple factors may explain the discrepancy in FAD/FADH_2_-associated respiration between the result of our work and those of a previous study 48. The mitochondrial respiration assay in the testing tube may destabilize CI-, CIII-, and CIV-containing supercomplexes in a time-dependent manner 14; thus, the difference in measurement conditions may explain the conflicting results.

CII acts as a key TCA cycle enzyme that dehydrogenates succinate into fumarate. It is plausible that CII inhibition may lead to the inhibition of the TCA cycle. A previous study revealed that CII inhibition suppressed of pyrimidine biosynthesis and negatively regulated other TCA-linked anabolism 57. Similar metabolic features have been observed in both the CII inhibition and COX7A2L depletion cell models. In accordance with our study, published work specifically highlight the importance of CII in glutamine-mediated TCA cycling 57. Likewise, our findings on the effects of CII inhibition on the downregulation of oxidative glutamine metabolism have been previously demonstrated 58. Although how CII promotes glutamine oxidation is not known, it is plausible to hypothesize that the rapid clearance of succinate by CII accelerates the TCA cycle and further promotes the entrance of glutamine into the cycle. Under this scenario, COX7A2L depletion may exert its antioxidant effect either through promoting the generation of antioxidant-metabolite aspartate or through accelerating the removal of succinate, which is necessary for the reverse electron transfer-mediated ROS accumulation 63. Notably, we did not observe a significant alteration of glucose metabolism in COX7A2L-deficient 293T cells, probably because glucose oxidation generates higher NADH/FADH_2_ ratio (5) 59, compared with glutamine oxidation (2-3) 64. While electrons from NADH and FADH_2_ enter the ETC by CI and CII, respectively, compared to glutamine oxidation, glucose oxidation with a higher NADH/FADH_2_ ratio may be affected to a lesser extent by CII inhibition or COX7A2L depletion. However, further studies are necessary to validate the role of CII in glutamine oxidation and to address the reason why glucose oxidation is less affected by supercomplex assembly. Importantly, in disagreement with our findings, among others 23, 48, a study showed that breast cancer cells overexpressing COX7A2L exhibited higher endogenous mitochondrial respiration as well as higher fumarate levels than control cells. This is probably the only cell type where COX7A2L overexpression has been reported to increase mitochondrial respiration 27. While COX7A2L was shown to have tissue-specific roles in mitochondrial supercomplex assembly, it is likely that COX7A2L may play different roles in the regulation of mitochondrial respiration and metabolic pathways 14. Moreover, the depletion of COX7A2L was shown the assembly of CIII, which is known to positively regulate cell proliferation by maintaining high anabolic metabolites levels 4, 48. Thus, it is likely that COX7A2L can also regulate cell metabolism via CIII assembly. The indispensable role of COX7A2L in the regulation of cell metabolism is also challenging because many differences observed in HEK293T cells and DBA mice, with and without COX7A2L, were relatively marginal. Altogether, these facts revealed that the regulatory roles of COX7A2L in metabolism are complicated and require further in-depth studies.

The regulatory role of mitochondrial supercomplexes in the growth of PDAC cells has not been previously reported. In accordance with other studies, our study proved that functional OXPHOS is essential for promoting the development of PDAC 53, 65. This could be partially attributed to the fact that an intact TCA cycle for PDAC cell proliferation requires OXPHOS 4, 6. Our study on PDAC adds to the existing literature on the mechanisms of glutamine addictive phenotypes, and for the first time, links mitochondrial supercomplex assembly to the regulation of glutaminolysis. Notably, during our study, supercomplexes were found to be important for the proliferation of PDAC cells under severe hypoxia 66. Kate E.R. Hollinshead et al. showed 66 that mitochondrial supercomplex, as well as its assembly factor COX7A2L, promotes glutamine-mediated oxidative metabolism for PDAC cell proliferation under severe hypoxia, whereas our study, found that COX7A2L promotes PDAC cell proliferation under glutamine deprivation, indicating that other uncharacterized metabolic pathways may be affected by supercomplex assembly. Although both our studies admit that COX7A2L promotes PDAC proliferation under stress, Kate E.R. Hollinshead et al. found 66 that COX7A2L is required for glutaminolysis while our study indicated that the loss of COX7A2L promoted glutaminolysis. In Hollinshead et al.'s study 66, cells were maintained under severe hypoxia, generating minimal levels of NAD^+^ and FAD from NADH and FADH_2_, respectively, via respiration. While both NADH and FADH_2_ were overloaded and oxygen availability was extremely low, compaction in the control of electron flux for CI and CII did not exist 23. Thus, respirasomes with high electron transfer efficiency may increase the rates of TCA cycle reactions and glutamine oxidation 10. In our study, however, electron flux preference was retained between CI and CII when oxygen was overloaded, with CII based electron flux further promoting glutaminolysis.

Since KRAS mutations are one of the major driving forces of glutaminolysis during the development of PDAC, and since our preliminary data suggested that COX7A2L regulated glutamine metabolism in PDAC, it is of great interest to uncover the relationship between KRAS activation and mitochondrial supercomplex assembly in the future. Furthermore, we believe that the physiological contribution of metabolic reprogramming due to COX7A2L-regulated supercomplex formation has major implications for human disease, not only because glutaminolysis drives the growth of cancer and the development of degenerative diseases 51, 67 but also because succinate accumulation due to supercomplex assembly or COX7A2L expression is an endogenous mitochondrial signal that initiates pathways involved in multiple processes such as adipose tissue thermogenesis and carcinogenesis 68, 69.

## Conclusion

In summary, we showed that COX7A2L promotes the assembly of mitochondrial supercomplexes and limits CII-dependent respiration. As an ON/OFF switch, the loss of COX7A2L in human cells enhanced CII-dependent respiration and reprogrammed metabolic pathways toward glutaminolysis-mediated anabolism. Our findings also highlighted the metabolic role of mitochondrial supercomplex assembly in human diseases such as PDAC.

## Supplementary Materials

Supplementary figures and table.
