# Supplementary material for: Mitochondrial supercomplex assembly regulates metabolic features and glutamine dependency in mammalian cells
Source: Theranostics. 2023 May 21;13(10):3165–87. doi: 10.7150/thno.78292 (PMC10283060; doi:10.7150/thno.78292)
Supplement: Supplementary file 1 — Supplementary figures and table. [file thnov13p3165s1.pdf]

Mitochondrial supercomplex assembly regulates metabolic features and glutamine dependency in mammalian cells

Kun Zhang[1,2#], Linjie Chen[4,5#], Bo Wang[1#], Deyu Chen[1#], Xianglai Ye[1], Xinyu Han[1], Quan Fang[4], Can Yu[1], Jia Wu[1], Sihan Guo[1], Lifang Chen[1], Yu Shi[1], Lan Wang[1], Huang Cheng[1], Hao Li[1], Lu Shen[1], Qiongya Zhao[3], Liqin Jin[3], Jianxin Lyu[1,3✉], Hezhi Fang[1,3✉]

# These authors contributed equally to this work

1. Key Laboratory of Laboratory Medicine, Ministry of Education, Zhejiang Provincial Key Laboratory of Medical Genetics, Department of Cell Biology and Medical Genetics, College of Laboratory Medicine and Life sciences, Wenzhou Medical University, Wenzhou 325035, China.

2. Department of Clinical Laboratory, Xi'an Daxing Hospital, Xi'an 710016, China.

3. Department of Laboratory Medicine, Zhejiang Provincial People's Hospital, Affiliated People's Hospital of Hangzhou Medical College, Hangzhou 310000, China.

4. School of Laboratory Medicine and Bioengineering, Hangzhou Medical College, Hangzhou 310053， China.

5. Key Laboratory of Biomarkers and In Vitro Diagnosis Translation of Zhejiang province, Zhejiang, Hangzhou, 310063, China.

✉Corresponding authors: **Prof. Jianxin Lyu**, Key Laboratory of Laboratory Medicine, Ministry of Education, Zhejiang Provincial Key Laboratory of Medical Genetics, Department of Cell Biology and Medical Genetics, College of Laboratory Medicine and Life sciences, Wenzhou Medical University, Wenzhou 325035, China. E-mail: jxlu313@163.com (J.L.) **Prof. Hezhi Fang**, Key Laboratory of Laboratory Medicine, Ministry of Education, Zhejiang Provincial Key Laboratory of Medical Genetics, Department of Cell Biology and Medical Genetics, College of Laboratory Medicine and Life sciences, Wenzhou Medical University, Wenzhou 325035, China.
E-mail: FangH@wmu.edu.cn (H.L.).

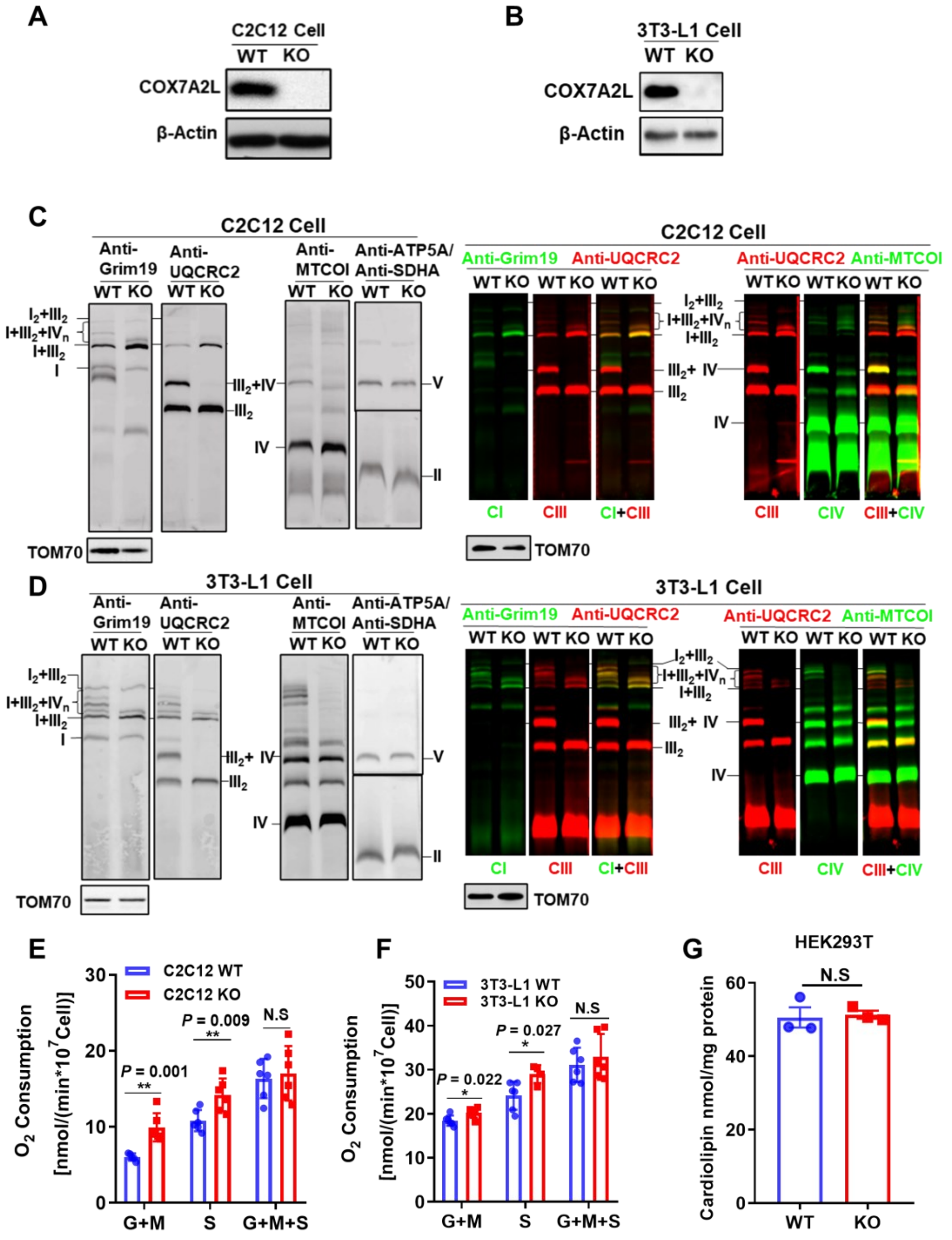

Figure S1. (**A-B**) Western blot analysis of COX7A2L in control (WT) and *COX7A2L*$^{-/-}$ (KO) C2C12 (**A**) and 3T3-L1 (**B**) cells. β-Actin was used as an internal control. (**C-D**) Blue native PAGE/immunoblotting analysis of respiratory chain supercomplexes in WT and KO C2C12 (**C**) and 3T3-L1 (**D**) cells. Mitochondria was solubilized with digitonin. Complex I-V were immunoblotted with anti-Grim19, anti-SDHA, anti-UQCRC2, anti-MTCOI, and anti-ATP5A antibodies, respectively. Alkaline phosphatase-conjugated or fluorescence-conjugated

secondary antibodies were applied. Samples were denatured and applied to an SDS-PAGE for incubation of anti-TOM70 as a loading control. (**E-F**) Glutamate and malate (G+M)-dependent, succinate (S)-dependent and glutamate, malate and succinate (G+M+S)-dependent respiration in WT and KO C2C12 (**E**) and 3T3-L1 cells (**F**) with 6 independent replicates. **(G)** Cardiolipin quantitation of HEK293T COX7A2L$^{+/+}$ and COX7A2L$^{-/-}$cells. Quantitative data are presented as mean ± SEM. N.S, not significant; * $P \leq 0.05$, ** $P \leq 0.01$.

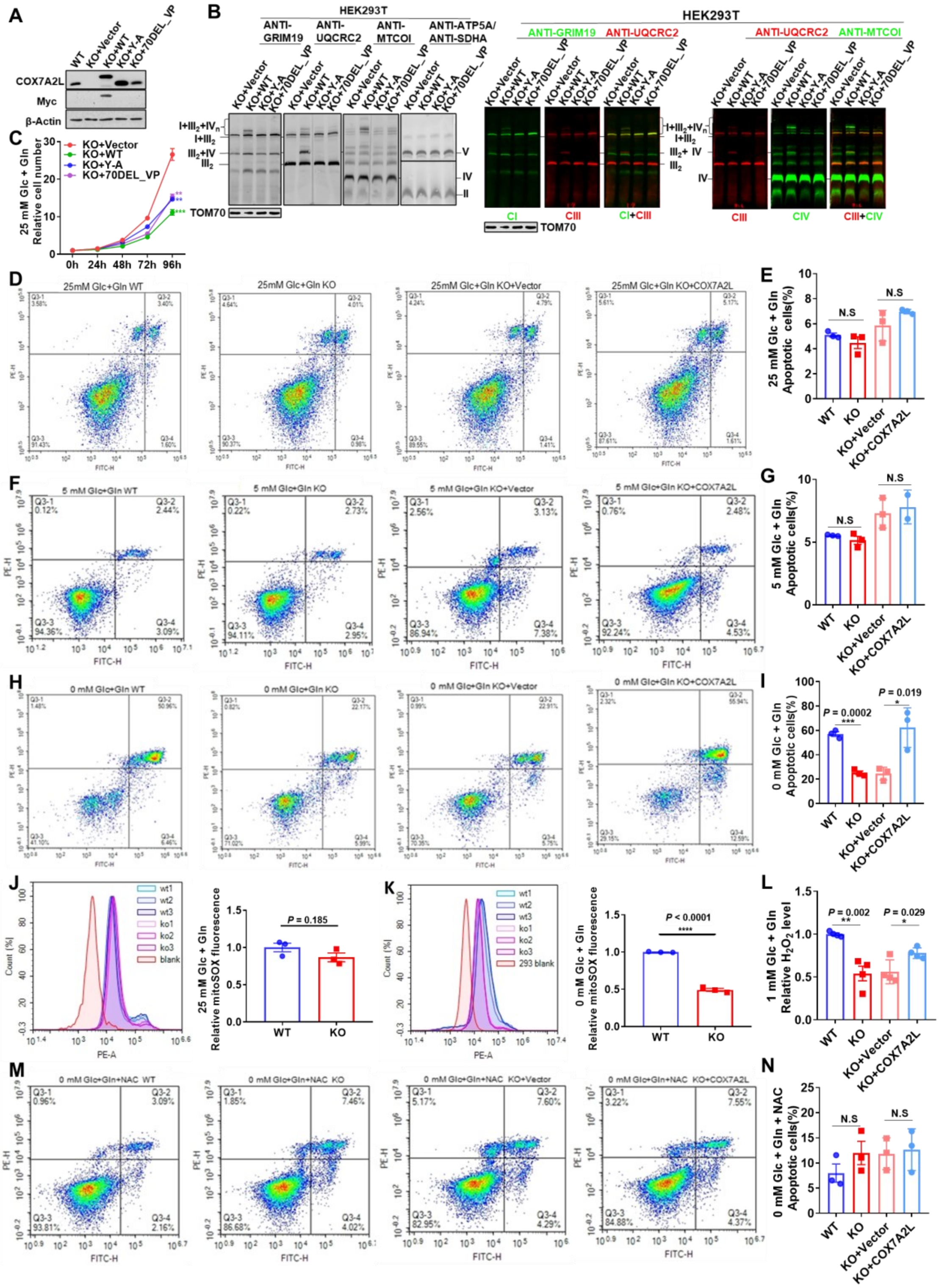
A
WT
KO+Vector
KO+WT
KO+Y-A
KO+70DEL_VP
COX7A2L
Myc
β-Actin
B
HEK293T
ANTI-GRIM19
ANTI-UQCRC2
ANTI-MTCOI
ANTI-ATP5A/ ANTI-SDHA
KO+Vector
KO+WT
KO+Y-A
KO+70DEL_VP
I+III2+IVn
I+III2
III2+IV
III2
V
IV
II
TOM70
HEK293T
ANTI-GRIM19
ANTI-UQCRC2
ANTI-UQCRC2
ANTI-MTCOI
CI
CIII
CI+CIII
CIII
CIV
CIII+CIV
TOM70
C
25 mM Glc + Gln
Relative cell number
KO+Vector
KO+WT
KO+Y-A
KO+70DEL_VP
0h
24h
48h
72h
96h
D
25mM Glc+Gln WT
25mM Glc+Gln KO
25mM Glc+Gln KO+Vector
25mM Glc+Gln KO+COX7A2L
PE-H
FITC-H
E
25 mM Glc + Gln
Apoptotic cells(%)
N.S
N.S
WT
KO
KO+Vector
KO+COX7A2L
F
5 mM Glc+Gln WT
5 mM Glc+Gln KO
5 mM Glc+Gln KO+Vector
5 mM Glc+Gln KO+COX7A2L
Q3-1 0.12%
Q3-2 2.44%
Q3-3 94.36%
Q3-4 3.09%
Q3-1 0.22%
Q3-2 2.73%
Q3-3 94.11%
Q3-4 2.95%
Q3-1 2.56%
Q3-2 3.13%
Q3-3 86.94%
Q3-4 7.38%
Q3-1 0.76%
Q3-2 2.48%
Q3-3 92.24%
Q3-4 4.53%
PE-H
FITC-H
G
5 mM Glc + Gln
Apoptotic cells(%)
N.S
N.S
WT
KO
KO+Vector
KO+COX7A2L
H
0 mM Glc+Gln WT
0 mM Glc+Gln KO
0 mM Glc+Gln KO+Vector
0 mM Glc+Gln KO+COX7A2L
PE-H
FITC-H
I
0 mM Glc + Gln
Apoptotic cells(%)
P = 0.0002
***
P = 0.019
*
WT
KO
KO+Vector
KO+COX7A2L
J
wt1
wt2
wt3
ko1
ko2
ko3
blank
Count (%)
PE-A
25 mM Glc + Gln
Relative mitoSOX fluorescence
P = 0.185
WT
KO
K
wt1
wt2
wt3
ko1
ko2
ko3
293 blank
Count (%)
PE-A
0 mM Glc + Gln
Relative mitoSOX fluorescence
P < 0.0001
****
WT
KO
L
1 mM Glc + Gln
Relative H2O2 level
P = 0.002
**
P = 0.029
*
WT
KO
KO+Vector
KO+COX7A2L
M
0 mM Glc+Gln+NAC WT
0 mM Glc+Gln+NAC KO
0 mM Glc+Gln+NAC KO+Vector
0 mM Glc+Gln+NAC KO+COX7A2L
Q3-1 0.96%
Q3-2 3.09%
Q3-3 93.81%
Q3-4 2.16%
Q3-1 1.85%
Q3-2 7.46%
Q3-3 86.68%
Q3-4 4.02%
Q3-1 5.17%
Q3-2 7.60%
Q3-3 82.95%
Q3-4 4.29%
Q3-1 3.22%
Q3-2 7.55%
Q3-3 84.88%
Q3-4 4.37%
PE-H
FITC-H
N
0 mM Glc + Gln + NAC
Apoptotic cells(%)
N.S
N.S
WT
KO
KO+Vector
KO+COX7A2L

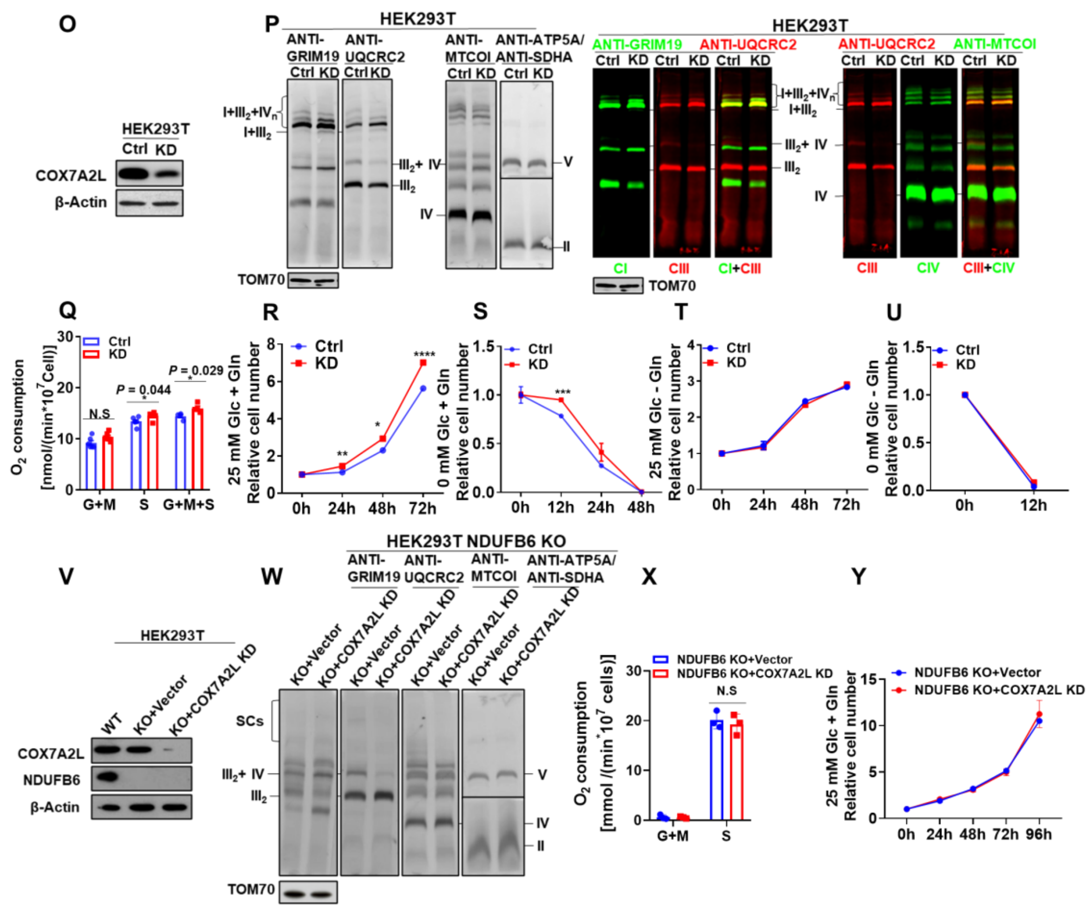

Figure S2. (**A**) Western blot analysis of COX7A2L and Myc Tag in 293T *COX7A2L*$^{-/-}$ cells (KO) expressing wild-type (KO+WT) and mutant COX7A2L (Y73A, Y-A; depletion of VP, 70DEL_VP). β-Actin was used as an internal control. (**B**) Blue native PAGE/immunoblotting analysis of respiratory chain supercomplexes in KO+Vector, KO+WT, KO+Y-A, and KO+70DEL_VP mitochondria solubilized with digitonin. Complex I-V were immunoblotted with anti-Grim19, anti-SDHA, anti-UQCRC2, anti-MTCOI, and anti-ATP5A antibodies, respectively. Alkaline phosphatase-conjugated or fluorescence-conjugated secondary antibodies were applied for different presentation of BNG. (**C**) Cell proliferation of KO cells with expression of vector, WT, Y-A, and 70DEL_VP mutant COX7A2L. (**D-I**) Apoptosis analysis of WT and KO as well as KO+Vector and KO+COX7A2L cells (n = 3). Cells were cultured in 25 mM medium (**D**), 5 mM glucose medium (**F**) or glucose-free medium (**H**) for 24 h. The apoptotic cells stained with both Annexin V-FITC and PI were counted (**E, G, I**). (**J-K**) Relative mitochondrial ROS of WT and KO cells (n = 3). Cells cultured in 25mM glucose (**J**) and glucose-free (**K**) medium for 24 h were stained by MitoSox for mitochondrial ROS determination. (**L**) Relative H_2O_2 level of 293T cells WT and KO as well as KO+Vector and KO+COX7A2L cells (n = 4) was cultured in 1 mM low-glucose medium for 48 h. (**M and N**) Apoptosis analysis of WT and KO as well as KO+Vector and KO+COX7A2L cells (n = 3) cultured in glucose-free medium containing 5 mM NAC for 24 h (**M**). The apoptotic cells

stained with both Annexin V-FITC and PI were counted (**N**). (**O**) Western blot analysis of COX7A2L in 293T transfected with pLKO.1 (Ctrl) and shCOX7A2L (KD). β-Actin was used as an internal control. (**P**) Blue native PAGE/immunoblotting analysis of respiratory chain supercomplexes in Ctrl and KD mitochondria solubilized with digitonin. Complex I-V were immunoblotted with anti-Grim19, anti-SDHA, anti-UQCRC2, anti-MTCOI, and anti-ATP5A antibodies, respectively. Alkaline phosphatase-conjugated or fluorescence-conjugated secondary antibodies were applied for different presentation of BNG. (**Q**) Glutamate and malate (G+M)-dependent, succinate (S)-dependent and glutamate, malate, and succinate (G+M+S)-dependent respiration in Ctrl and KD cells (n = 4). (**R-U**) Cell proliferation of Ctrl and KD cells cultured in 25 mM glucose (**R**), glucose-free (**S**), glutamine-free (**T**) and glucose glutamine-free (**U**) medium. (**V**) Western blot analysis of COX7A2L and NDUFB6 in 293T cells (WT) and NDUFB6$^{-/-}$ cells (KO) infected with lentivirus containing an empty vector (KO+Vector) or shCOX7A2L (KO+COX7A2L KD). β-Actin was used as an internal control. (**W**) Blue native PAGE/immunoblotting analysis of respiratory chain supercomplexes in NDUFB6 KO+Vector and KO+COX7A2L KD cells mitochondria solubilized with digitonin. Complex I, III and IV were immunoblotted with anti-Grim19, anti-UQCRC2, and anti-MTCOI antibodies, respectively. (**X**) Glutamate and malate (G+M)-dependent (n = 1) and succinate (S)-dependent (n = 5) respiration in NDUFB6 KO+Vector and KO+COX7A2L KD cells. (**Y**) Cell proliferation of NDUFB6 KO+Vector and KO+COX7A2L KD cells cultured in 25mM glucose medium. For cell proliferation analysis, cells were counted at indicated time point for three independent replicates and cell number was normalized to the initial time point (0 h) when cells were seeded. For blue native PAGE, samples were denatured and applied to SDS-PAGE for incubation of anti-TOM70 as a loading control. Quantitative data are represented as mean ± SEM. N.S, not significant; *$P \leq 0.05$, **$P \leq 0.01$, ***$P \leq 0.001$, ****$P \leq 0.0001$.

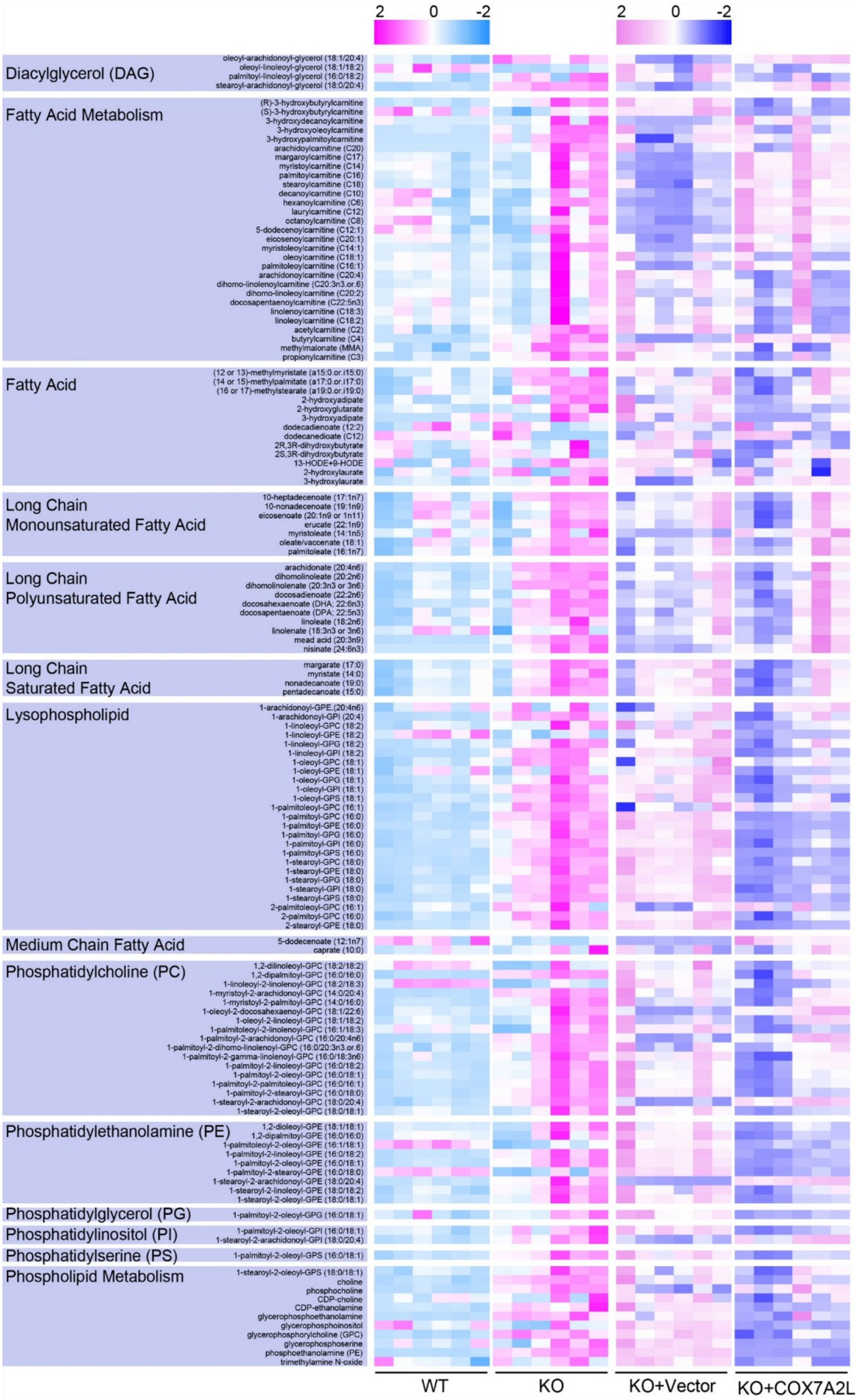

Figure S3. Heat map of fatty acid metabolism in WT and KO as well as KO+Vector and KO+COX7A2L cells (n = 6). Data were obtained from untargeted metabolomics. Comparison was done between WT and KO cells as well as between KO+Vector and KO+COX7A2L cells.

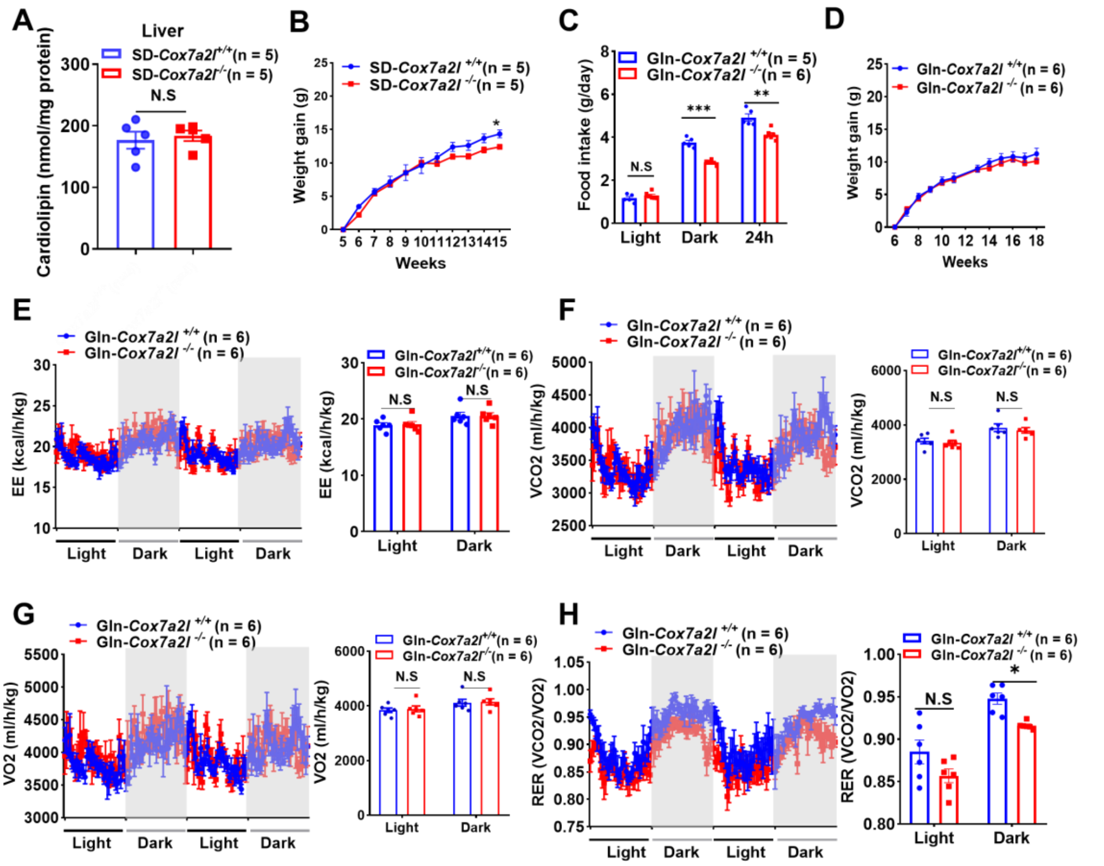

Figure S4. (**A-B**) Liver cardiolipin quantitation (**A**) and gain of body weight (**B**) of *Cox7a2l*$^{+/+}$ and *Cox7a2l*$^{-/-}$ mice fed with standard diet (SD). (**C-H**) Food intake (**C**), gain of body weight (**D**), energy expenditure (EE, **E**), carbon dioxide production (VCO_2, **F**), oxygen consumption (VO_2, **G**), and respiratory exchange ratio (RER, **H**) of *Cox7a2l*$^{+/+}$ and *Cox7a2l*$^{-/-}$ mice fed with standard diet and glutamine supplementation (Gln). Quantitative data are presented as the mean ± SEM. N.S, not significant; *$P \leq 0.05$, **$P \leq 0.01$, ***$P \leq 0.001$.

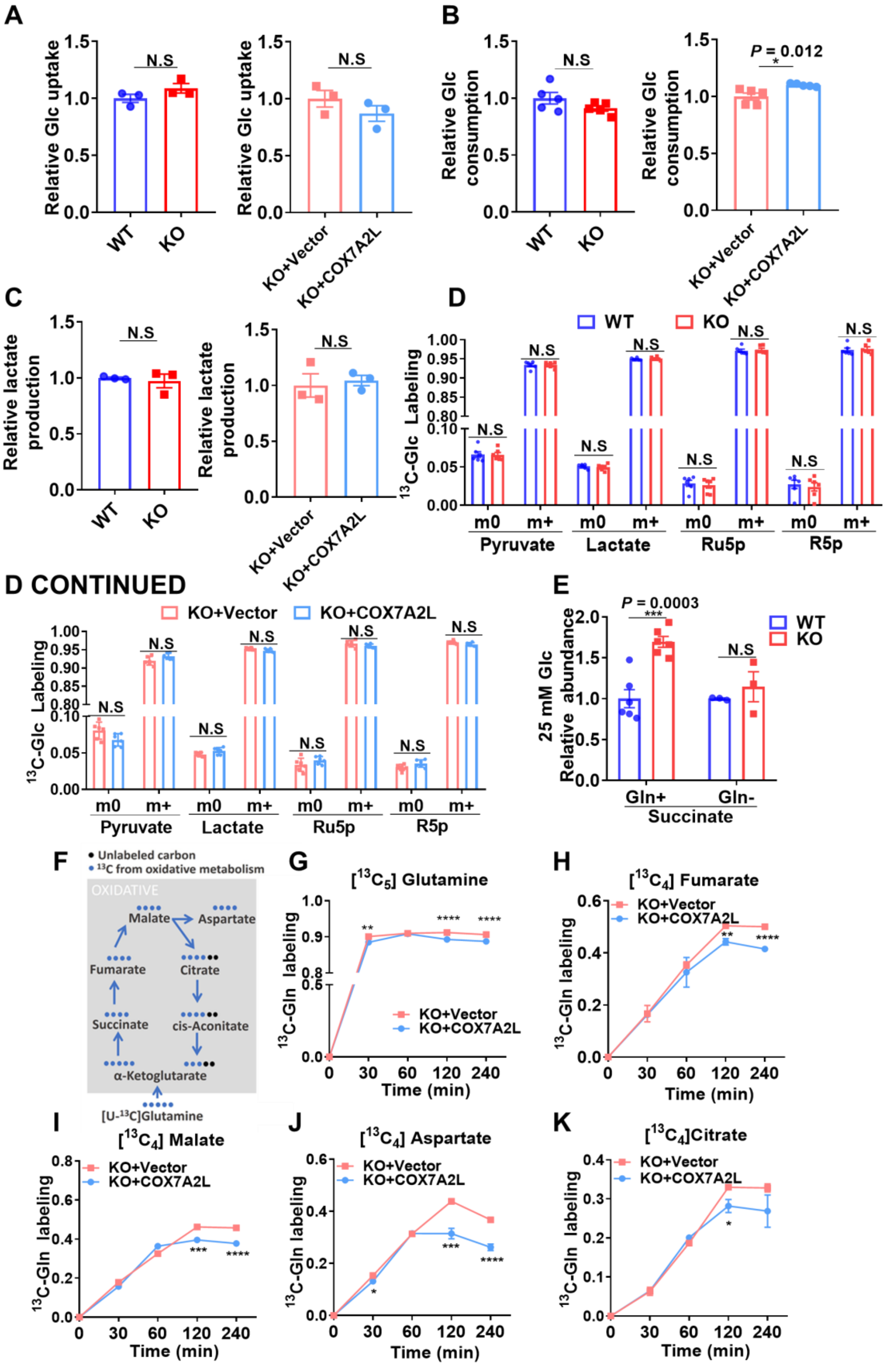

Figure S5. (**A-C**) Relative glucose uptake (n = 3, **A**), glucose consumption (n = 5, **B**), and lactate production (n = 3, **C**) in WT and KO as well as KO+Vector and KO+COX7A2L cells. (**D**) $^{13}C_6$-glucose metabolic flux analysis of glycolysis and pentose phosphate pathway (PPP) metabolites in WT and KO as well as KO+Vector and KO+COX7A2Lcells (n = 6) after

incubating with $^{13}C_6$-glucose labeling medium for 18 h. m0, unlabeled metabolites; m+, labeled metabolites. **(E)** Relative succinate abundance in KO and WT cells cultured in presence (n = 6) or absence of glutamine (n = 3) for 24 h. Data was obtained from targeted metabolic analysis. **(F)** Schematic diagram of $^{13}C_5$-glutamine flux into TCA cycle. Intermediates from TCA cycle was shown with different isotope-labeling pattern. (**G-K**) $^{13}C_5$-glutamine metabolic flux analysis of labeled glutamine (**G**), fumarate (**H**), malate (**I**), aspartate (**J**), and citrate (**K**) abundance in KO+Vector and KO+COX7A2Lcells (n = 6) after incubating with $^{13}C_5$-glutamine labeling medium for different time periods. Quantitative data are represented as mean ± SEM. N.S, not significant; $*P \leq 0.05$, $**P \leq 0.01$, $***P \leq 0.001$, $****P \leq 0.0001$.

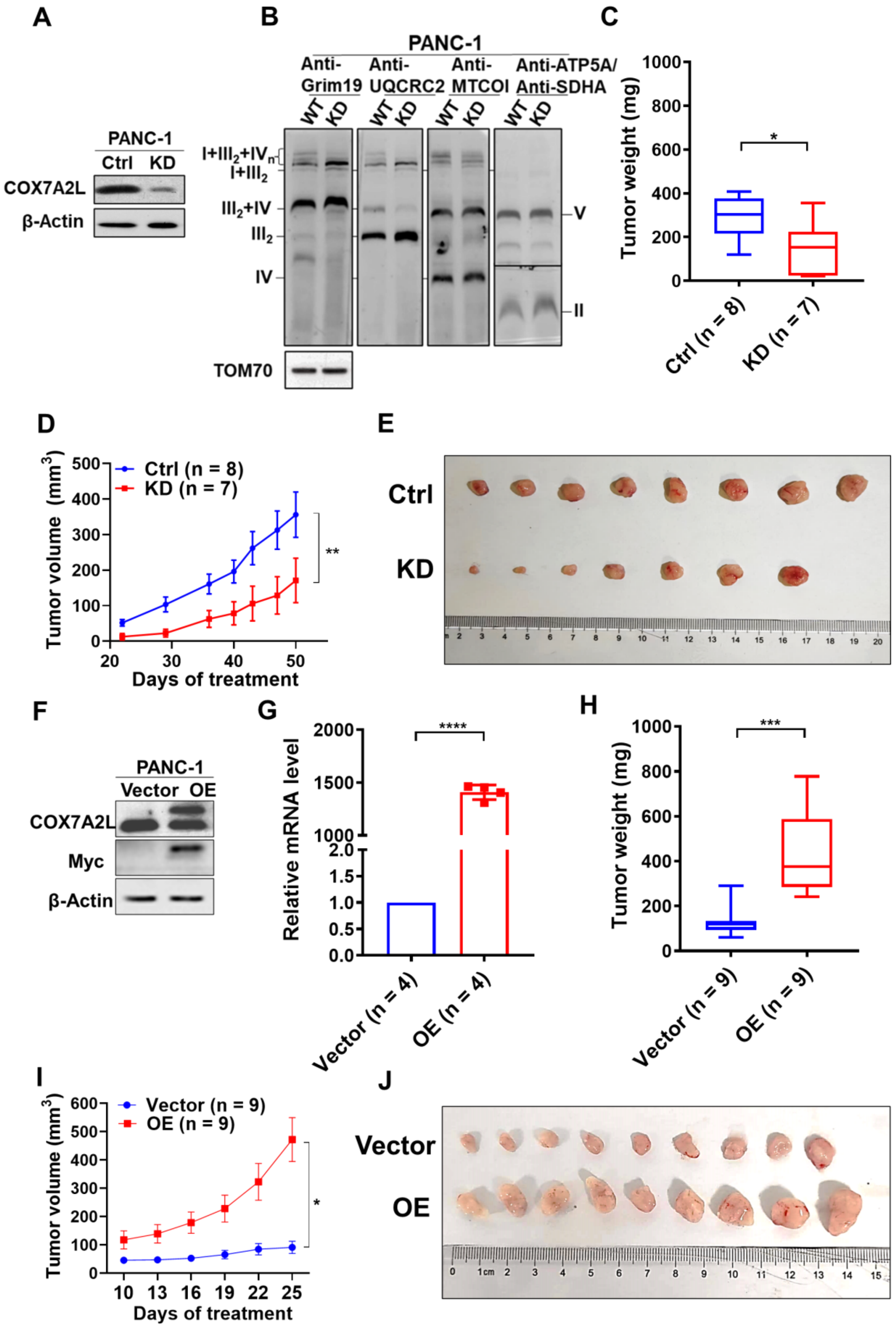

Figure S6. (A and F) Western blot analysis of COX7A2L in PANC-1 cells treated with control (Ctrl) or COX7A2L shRNA (KD) **(A)** and analysis of COX7A2L and Myc Tag in PANC-1 cells treated with control cells infected with lentivirus containing an empty vector (Vector) or

COX7A2L cDNA (OE) **(F).** β-Actin was used as an internal control. **(B)** Blue native PAGE of respiratory chain supercomplexes in control and KD PANC-1 cells. **(C-E, H-J)** Tumor weight (**C and H**) and tumor volume (**D and I**) in nude mice with subcutaneous injection of PANC-1 cells (5×10^6) in control or KD of COX7A2L for 8 weeks as well as vector or OE of COX7A2L for 4 weeks. 8 (**E**)or 9 (**J**) pairs of tumors were shown in the image. (**G**) qPCR validation of vector or COX7A2L OE in PANC-1 cells.

Table S1. Nutrient-conditioned medium for cell proliferation.

Conditioned	Glucose	Glutamine	Pyruvate	Calf serum	Penicillin	Streptomycin	Amphotericin B	Medium
25 mM Glc+Gln	--	--	--	12%[a]	100	100	0.25	DMEM[c]
5 mM Glc+Gln	5	4	0	12%[b]	100	100	0.25	DMEM[d]
1 mM Glc+Gln	1	4	0	12%[b]	100	100	0.25	DMEM[d]
0mM Glc+Gln	0	4	0	12%[b]	100	100	0.25	DMEM[d]
25 mM Glc-Gln	25	0	0	12%[b]	100	100	0.25	DMEM[d]
5 mM Glc-Gln	5	0	0	12%[b]	100	100	0.25	DMEM[d]
1 mM Glc-Gln	1	0	0	12%[b]	100	100	0.25	DMEM[d]
0 mM Glc-Gln	0	0	0	12%[b]	100	100	0.25	DMEM[d]

"--" indicates not add.

[a] Calf serum

[b] Dialyzed calf serum.

[c] High-glucose Dulbecco's modified Eagle's medium (DMEM, Sigma-Aldrich).

[d] Glutamine, glucose and pyruvate free medium (DMEM, Gibco)
